# Extractable Potentially Toxic Elements Across Soil Profiles in East Kazakhstan: Depth Patterns and Soil-Property Associations

**DOI:** 10.3390/toxics14100918

**Published:** 2026-10-16

**Authors:** Zhanar Abilda, Dias Daurov, Rakhim Kanat, Iskander Isgandarov, Zagipa Sapakhova, Ainash Daurova, Maxat Toishimanov, Kabyl Zhambakin, Malika Shamekova

**Affiliations:** 1 Institute of Plant Biology and Biotechnology, Almaty 050040, Kazakhstan; z.abilda@ipbb.kz (Z.A.); r.kanat@ipbb.kz (R.K.); i.isgandarov@ipbb.kz (I.I.); z.sapakhova@ipbb.kz (Z.S.); m.toishmanov@ipbb.kz (M.T.); k.zhambakin@ipbb.kz (K.Z.); 2 Tanir Research Laboratory, Almaty 050010, Kazakhstan

**Keywords:** potentially toxic elements (PTEs), East Kazakhstan Region, ammonium acetate extraction, vertical soil profiles, polymetallic mining, soil texture, extractable metal pool

## Abstract

The vertical distribution of operationally extractable potentially toxic elements may vary substantially among soil profiles, limiting the value of surface-only assessment. This study examined ammonium acetate buffer (AAB)-extractable Zn, Cu, Cd, and Pb in 486 soil samples from 133 profiles in the East Kazakhstan Region. Samples were obtained from four depth intervals: 0–5, 5–20, 20–50, and 50–100 cm. Soil-property analyses as core properties were assessed across 0–100 cm, nutrients across 0–50 cm, and humus and exchangeable cations within 0–20 cm. Depth-related patterns and associations with soil properties were evaluated using non-parametric tests, Spearman correlations, linear mixed-effects models, redundancy analysis and pooled percentile screening. Metal concentrations were strongly right-skewed and varied markedly among profiles. From 0–5 to 50–100 cm, median concentrations declined from 55.2 to 4.5 mg kg^−1^ for Zn, 5.7 to 1.7 mg kg^−1^ for Cu, 2.5 to 1.4 mg kg^−1^ for Cd, and 3.9 to 1.2 mg kg^−1^ for Pb. Depth-only mixed-effects models confirmed negative average depth trends for all four metals, alongside substantial between-profile heterogeneity. Sand content showed positive associations with all four metals across the analysed depth scopes; available P_2_O_5_ was positively associated with all four in the 0–50 cm models, while humus was positively associated with Zn, Cd, and Pb in the upper 20 cm. Observations at or above the pooled P90 and P95 thresholds were most frequent near the surface, although P95-level observations also occurred at 50–100 cm. These findings show that depth-related median declines coexist with substantial profile-specific heterogeneity and support depth-resolved monitoring of AAB-extractable metals in polymetallic mining and metallurgical landscapes.

## 1. Introduction

Potentially toxic elements (PTEs) are among the most persistent chemical contaminants in terrestrial environments [1,2]. Unlike many organic pollutants, PTEs do not degrade and may remain in soils for prolonged periods [3,4]. Their environmental significance depends not only on their overall occurrence in soil but also on their distribution among operationally defined soil fractions and the proportion present in extractable forms that can participate in soil geochemical processes. The accumulation of PTEs can alter soil geochemical conditions [5], while their occurrence in operationally extractable forms is relevant to their potential redistribution within soil systems and interactions with soil organisms, vegetation and water [6,7,8,9]. Zinc (Zn) and copper (Cu) are essential micronutrients but can become phytotoxic or ecotoxic at elevated concentrations [10,11,12], whereas cadmium (Cd) and lead (Pb) have no known biological requirement and are recognised for their toxicological relevance [13,14].

East Kazakhstan Region is one of the major mining and metallurgical areas of Central Asia and contains numerous polymetallic deposits associated with Zn, Pb, Cu, Cd and other sulphide-bearing ores [15,16]. The region has a long history of ore extraction, beneficiation and non-ferrous metallurgical production [17,18]. Mining, ore processing, tailings storage, industrial emissions, and the redistribution of metal-bearing dust may contribute to the accumulation and spatial redistribution of PTEs in surrounding soils. The resulting contamination patterns can be highly heterogeneous because metal inputs interact with differences in soil properties and landscape position.

Ammonium acetate buffer (AAB) extraction provides an operational measure of a relatively weakly bound metal pool that may respond differently to soil conditions than total metal concentrations. Focusing on AAB-extractable Zn, Cu, Cd, and Pb therefore helps identify variation in this pool across soil profiles and provides a consistent basis for examining its associations with depth and soil properties [19,20].

Vertical differentiation is a central feature of soil systems [21]. Soil properties that influence PTE retention and extractability-such as pH, organic matter, carbonate content, nutrient status, and exchange conditions-can vary substantially with depth [22,23]. Surface horizons can receive atmospheric deposition and other anthropogenic inputs [24] and commonly contain higher concentrations of organic matter and biologically active constituents [25]. With increasing depth, changes in soil reaction, carbonate status and mineral composition can modify the retention and extractability of PTEs [26].

The interpretation of depth-related PTE patterns requires statistical approaches that account for both vertical structure and local heterogeneity. Comparisons among depth intervals reveal broad stratification [27], while mixed-effects models can estimate average depth gradients after accounting for non-independence of samples collected within profiles [28]. Multivariate analyses can further identify common patterns among co-occurring metals and evaluate their relationships with soil physicochemical properties [29]. Integrating these approaches can therefore distinguish the general vertical structure of extractable PTE concentrations from profile-specific variation and identify the soil conditions associated with elevated metal pools.

From an environmental-safety perspective, identifying soil-profile intervals with elevated extractable metal concentrations is important for risk-oriented site characterization and for prioritizing monitoring [30]. Vertical metal distributions provide an important basis for evaluating contamination, migration, and remediation needs in affected soils [31]. Screening for unusually high observations can complement conventional summary statistics by highlighting localized concentration anomalies that may be masked by pronounced spatial heterogeneity [30,32]. Such screening is particularly relevant in contaminated landscapes where elevated concentrations may occur both near the surface and, less frequently, in deeper soil layers [33].

The present study investigated AAB-extractable Zn, Cu, Cd, and Pb across four soil-depth intervals (0–5, 5–20, 20–50, and 50–100 cm). The objectives were to: (i) characterise the depth-resolved distribution of AAB-extractable metal concentrations; (ii) test differences among depth intervals; (iii) estimate average depth–metal relationships while accounting for clustering of observations within soil profiles; (iv) evaluate associations between the multivariate metal pattern and selected soil physicochemical properties. We hypothesised that AAB-extractable metal concentrations would generally decline with increasing depth, that the magnitude of vertical gradients would differ among metals, and that variation in extractable metal concentrations would reflect both depth-dependent changes in soil properties and substantial profile-specific heterogeneity.

## 2. Materials and Methods

### 2.1. Study Area and Sampling Design

**Figure 1:**
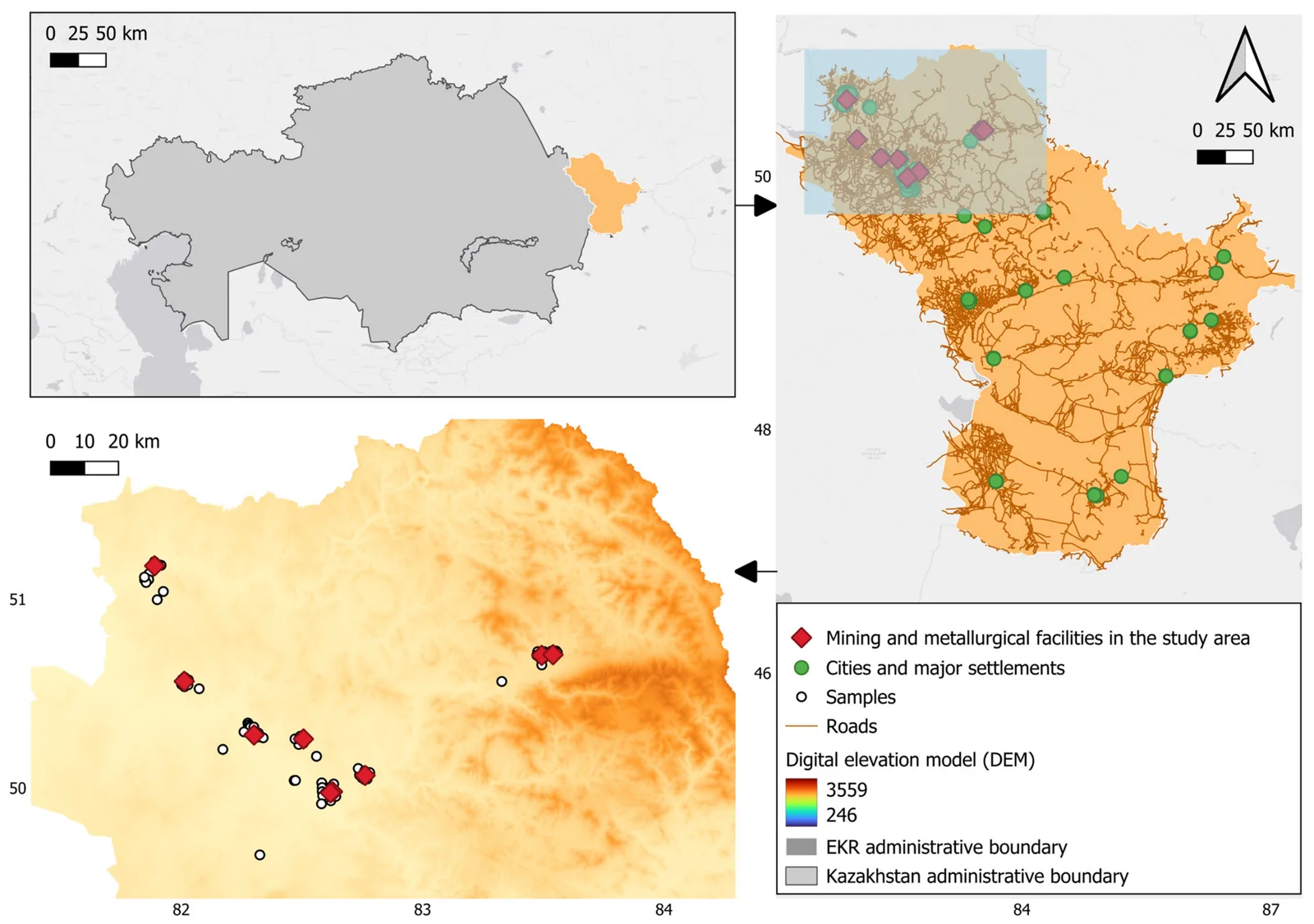
Study area and sampling locations.

The study was conducted in East Kazakhstan Region (Figure 1). The region covers approximately 97.9 thousand km^2^ and includes strongly contrasting geomorphological settings. The eastern and northeastern parts are dominated by the Altai mountain system and associated highland landscapes, whereas central and western areas are characterized mainly by plains with steppe, semi-desert, and desert landscapes. This topographic contrast is accompanied by substantial variation in soil-forming conditions, hydrology, and the potential redistribution of fine particles and metal-bearing material across the landscape.

The regional climate is sharply continental and ranges from semi-arid to arid, with pronounced seasonal and spatial variability. Mean annual air temperature ranges approximately from −2 to 5 °C, while annual precipitation varies from about 195 to 637 mm, with higher precipitation in the mountainous east and lower precipitation in the western lowlands [34]. The hydrographic network is dominated by the Irtysh River and its tributaries, including the Bukhtarma, Kurchum, Ulba, and Uba rivers. The region also contains major water bodies, including Lakes Zaysan and Markakol and the Bukhtarma and Ust-Kamenogorsk reservoirs.

Soil cover is diverse and reflects the marked gradients in elevation, climate, parent material, and land use. Under the genetic soil classification traditionally used in Kazakhstan, the regional soil cover includes chernozems, chestnut soils, forest soils, brown soils, mountain soils, and solonchaks. In broad World Reference Base terms, these groups correspond approximately to Chernozems, Kastanozems, Cambisols and/or Leptosols, Calcisols and Solonchaks [35]. Within the Ust-Kamenogorsk area, dark chestnut soils, mountain chernozems, meadow-forest soils, and alluvial meadow soils are common; these correspond broadly to Kastanozems, Chernozems, and Fluvisols.

East Kazakhstan Region is one of Kazakhstan’s principal mining and non-ferrous metallurgical regions and contains numerous polymetallic ore deposits associated with Zn, Pb, Cu, Cd, and other sulphide-bearing minerals. The sampled area includes industrially influenced landscapes associated with the Ust-Kamenogorsk Metallurgical Complex, including zinc, lead, precious-metal refining, and sulphuric-acid production facilities, as well as the Ust-Kamenogorsk Titanium–Magnesium Plant. The regional industrial setting also includes the Belousovsky mining and concentrating complex, the Irtysh mining and processing facilities, the Irtysh copper-smelting plant, the Irtysh rare-earth-metals plant, and the Nikolayevsky mining and concentrating facilities. In the Ridder area, major facilities include the Ridder–Sokolny mining complex and the Ridder concentrator/zinc-production facilities. These enterprises process or have historically processed polymetallic ores and concentrates and are associated with mining, ore beneficiation, smelting, refining, and related industrial activities.

The Belousovsky, Irtyshsky, and Nikolayevsky facilities are listed among the mining and concentrator operations of East Kazakhstan in U.S. Geological Survey mineral-industry reports. The USGS also identifies the Ridder–Sokolny mine, the Ridder concentrator, and the Ridder zinc refinery as important facilities in the region [36].

Two analytical campaigns were conducted for this study. The first campaign, carried out in 2024, included soil sampling across the East Kazakhstan Region and analysis of soil physicochemical properties. Sampling locations were selected to represent soils within industrially influenced areas associated with major mining and metallurgical facilities, as well as surrounding locations. The second campaign, conducted in May 2025, determined AAB-extractable Zn, Cu, Cd, and Pb concentrations in the same soil samples. The survey comprised 133 soil profiles and 486 soil samples. Each sample represented an individual field sample collected from the designated profile and depth interval; no composite samples were used.

**Figure 2:**
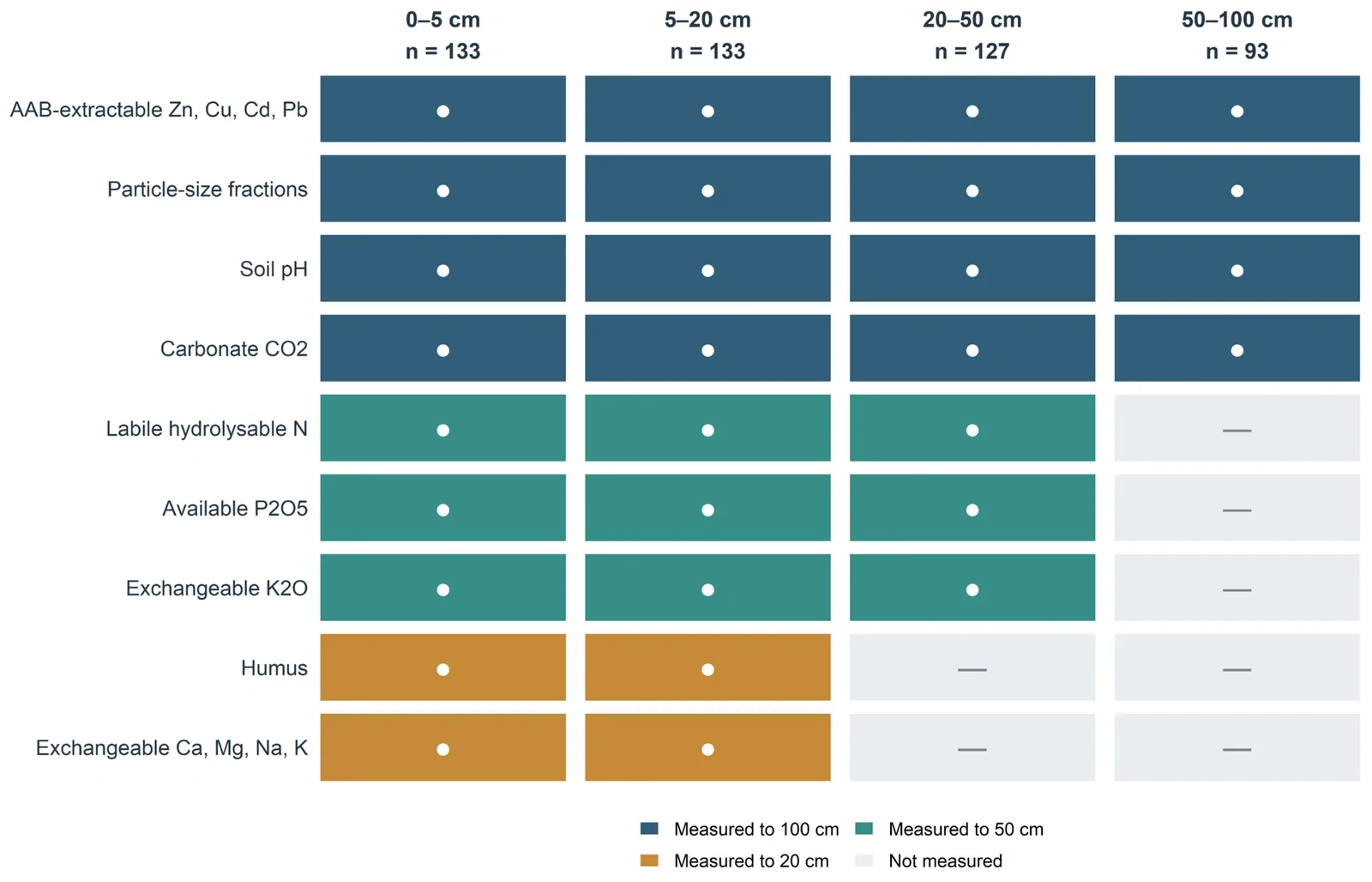
Number of soil samples analysed at each depth interval. Note: Dots indicate that the corresponding soil parameter was measured at the specified depth interval; dashes (—) indicate that it was not measured. Exchangeable cations were not determined for one sample in each of the 0–5 and 5–20 cm depth intervals; therefore, *n* = 132 for each element in each interval.

Consequently, sample availability decreased with depth: 133 samples were obtained from both the 0–5 and 5–20 cm intervals, 127 samples from 20 to 50 cm, and 93 samples from 50 to 100 cm. Sampling was terminated where further penetration was prevented by bedrock or coarse rock fragments. The analytical programme included AAB-extractable Zn, Cu, Cd, and Pb, pH, carbonate CO_2_, and particle-size fractions for all collected samples. The determination of humus, labile hydrolysable nitrogen, available P_2_O_5_, exchangeable K_2_O, and exchangeable bases was limited to selected depth intervals as part of the original laboratory analytical programme: humus and exchangeable bases were measured at 0–5 and 5–20 cm, whereas nitrogen, available P_2_O_5_, and exchangeable K_2_O were measured to 50 cm. These variables were therefore analysed only within the depth ranges for which measurements were available; no values were imputed or extrapolated to deeper intervals. The number of samples analysed for each measured property at each depth interval is shown in Figure 2.

Following collection, samples were placed in clean labelled cloth bags and transported to the laboratory. Samples were air-dried under laboratory conditions, gently disaggregated, and passed through a 100-mesh sieve (150 μm) before chemical analysis. Where replicate field samples were collected, they were treated as independent quality-control samples and used to assess sampling reproducibility.

### 2.2. Soil Chemical Properties

Total humus content was determined by the Tyurin method according to GOST 26213-91 [37]. The method is based on oxidation of humus with potassium dichromate in sulfuric acid medium followed by back-titration of excess dichromate with Mohr’s salt. Results were expressed as percent of dry soil. Labile hydrolysable nitrogen was determined by the Tyurin-Kononova method. Nitrogen was extracted with 0.5 N H_2_SO_4_, hydrolysed for 24 h, and the released ammonia was distilled into boric acid after alkalisation and quantified by titration with standard acid. Results were expressed in mg kg^−1^. Available phosphorus and exchangeable potassium in carbonate soils were determined by the Machigin method using 1% ammonium carbonate solution at pH 9.0. Phosphorus was quantified colorimetrically as a blue phosphomolybdate complex, while potassium was determined by flame photometry. Results were reported as available P_2_O_5_ and exchangeable K_2_O (mg kg^−1^), respectively, following GOST 26205-91 [38].

Soil pH was measured potentiometrically after calibration with standard buffer solutions of pH 4.01, 6.86, and 9.18. The total measurement uncertainty did not exceed 0.1 pH units. Carbonate content was determined as carbonate CO_2_ by the H.P. Bauer method. The method is based on acid decomposition of soil carbonates and quantification of evolved CO_2_. Carbonate CO_2_ was expressed as a percent of soil and used as an indicator of carbonate status.

### 2.3. Determination of AAB-Extractable Metals

Mobile Zn, Cu, Cd, and Pb compounds were extracted from soil using an ammonium acetate buffer solution (AAB) at pH 4.8 in accordance with RD 52.18.289-90 [39]. Air-dried soil samples were extracted with AAB at a soil-to-solution ratio of 1:10. A 5 g soil aliquot was mixed with 50 mL of the extractant and kept for 24 h at room temperature with intermittent mixing. The suspensions were subsequently filtered through a folded ashless paper filter with a particle-retention range of 2–3 μm and an ash mass of no more than 0.0010 g per filter. Zn, Cu, Cd, and Pb concentrations in the filtrates were determined by flame atomic absorption spectrometry (FAAS) using an AA-6200 spectrometer (Shimadzu, Kyoto, Japan). Results were expressed as mg kg^−1^ dry soil. Calibration standards of 0.5, 1.0, and 2.0 mg L^−1^ were used for Cu, Zn, and Pb, whereas standards of 0.10, 0.25, and 0.50 mg L^−1^ were used for Cd. A 25 mL aliquot of ammonium acetate buffer solution at pH 4.8 was used as the reagent blank.

Under these extraction conditions, ammonium ions primarily displace metals from readily exchangeable sites, while the mildly acidic buffer promotes desorption and acetate may contribute to the release of some weakly bound metal complexes.

### 2.4. Data Processing and Statistical Analysis

Data processing and statistical analyses were conducted in R 4.4.2. The laboratory datasets were merged by sample number.

Descriptive statistics included the number of available observations (*n*), arithmetic mean, standard deviation, median, median absolute deviation, interquartile range, minimum, maximum, coefficient of variation, and geometric mean. The value of *n* was reported separately for each variable and depth interval. Metal concentrations were log_10_-transformed before mixed-effects modelling and multivariate analyses because their distributions were strongly right-skewed and spanned wide concentration ranges:

(1) $$Y_{\mathrm{mi}}=\log_{10\left(C_{\mathrm{mi}}\right)}$$

where *Y_mi_* is the transformed concentration of metal *m* in sample *i*, and *C_mi_* is its observed concentration in mg kg^−1^. Descriptive statistics, non-parametric depth comparisons, Spearman correlations, and percentile thresholds were calculated on the original concentration scale.

Differences among depth intervals were assessed using Kruskal–Wallis tests followed by Dunn pairwise comparisons with Holm adjustment. Spearman rank correlations were calculated between AAB-extractable metal concentrations and soil predictors; *p*-values were adjusted separately for each metal using the Benjamini–Hochberg procedure. Linear mixed-effects models were fitted separately for AAB-extractable Zn, Cu, Cd, and Pb, using log_10_-transformed concentrations as responses and sampling site as a random intercept. Soil-property models were fitted for three depth scopes according to data availability: 0–100 cm (486 observations), 0–50 cm (393), and 0–20 cm (266). The 0–100 cm model included depth, pH, carbonate CO_2_, and sand; the 0–50 cm model additionally included nitrogen, available P_2_O_5_, and exchangeable K_2_O; and the 0–20 cm model additionally included humus, exchangeable Ca, Na, K and Mg. Sand was selected as the sole particle-size predictor after comparison with silt and the <0.001 mm fraction. Within each scope, prespecified nested models were fitted to the same complete-case observations by maximum likelihood and compared using AIC and likelihood-ratio tests.

Exploratory RDA used standardised metal responses and soil predictors; overall models, axes, and marginal terms were assessed with 999 permutations restricted within sampling profiles.

## 3. Results and Discussion

### 3.1. Descriptive Statistics of Soil Physicochemical Properties

**Figure 3:**
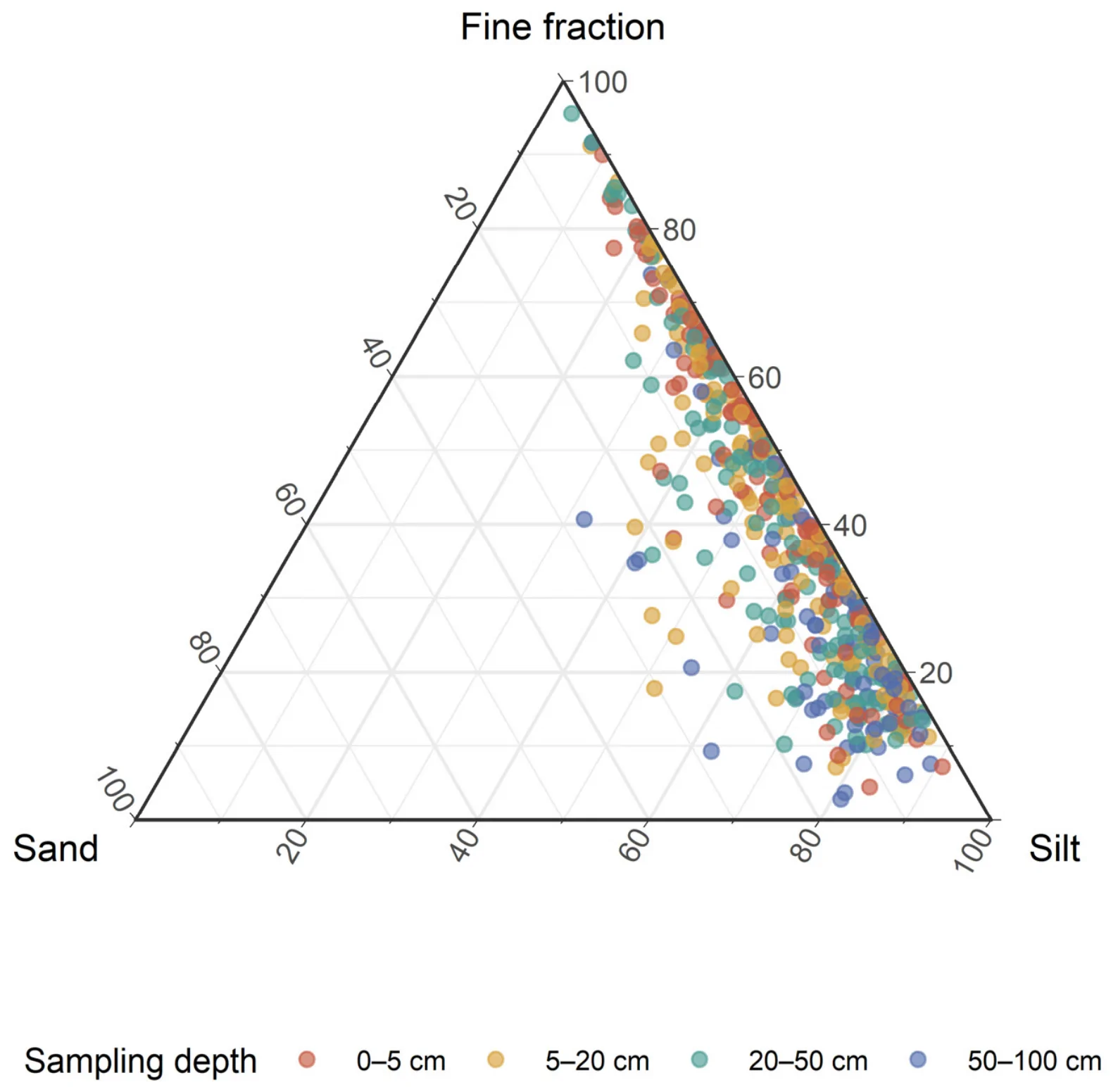
Soil particle-size composition triangle for the analysed samples.

Descriptive statistics for soil properties by depth interval are presented in Supplementary Table S1. Soil pH increased modestly with depth: median pH values were 6.57, 6.65, 6.86, and 7.03 at 0–5, 5–20, 20–50, and 50–100 cm, respectively. Median humus decreased from 4.17% in the 0–5 cm layer to 3.48% at 5–20 cm; humus was unavailable for deeper layers. Median labile hydrolysable nitrogen decreased from 58.8 mg kg^−1^ at 0–5 cm to 50.4 mg kg^−1^ at 5–20 cm and 44.8 mg kg^−1^ at 20–50 cm. Median available P_2_O_5_ declined from 50 to 30 and 16 mg kg^−1^ across these depth intervals, while median exchangeable K_2_O declined from 440 to 370 and 230 mg kg^−1^. Median carbonate CO_2_ was 0.42%, 0.40%, 0.515%, and 0.53% across the four depth intervals. In the upper-profile subset, median sums of measured exchangeable bases were 22.24 and 22.17 meq 100 g^−1^ in the 0–5 and 5–20 cm layers, respectively, and Ca was the dominant exchangeable cation. Soil particle-size composition varied with depth. Median sand content decreased from 44.24% at 0–5 cm to 38.86%, 34.33%, and 24.68% at 5–20, 20–50, and 50–100 cm, respectively. Conversely, median silt content increased from 52.33% in the surface layer to 54.58%, 61.17%, and 71.06% across the successive depth intervals. The median fine fraction (<0.001 mm) increased from 2.05% at 0–5 cm to 3.24% at 5–20 cm and 4.46% at 20–50 cm, before declining slightly to 3.67% at 50–100 cm (Figure 3).

### 3.2. Depth Distribution of AAB-Extractable Metals

**Figure 4:**
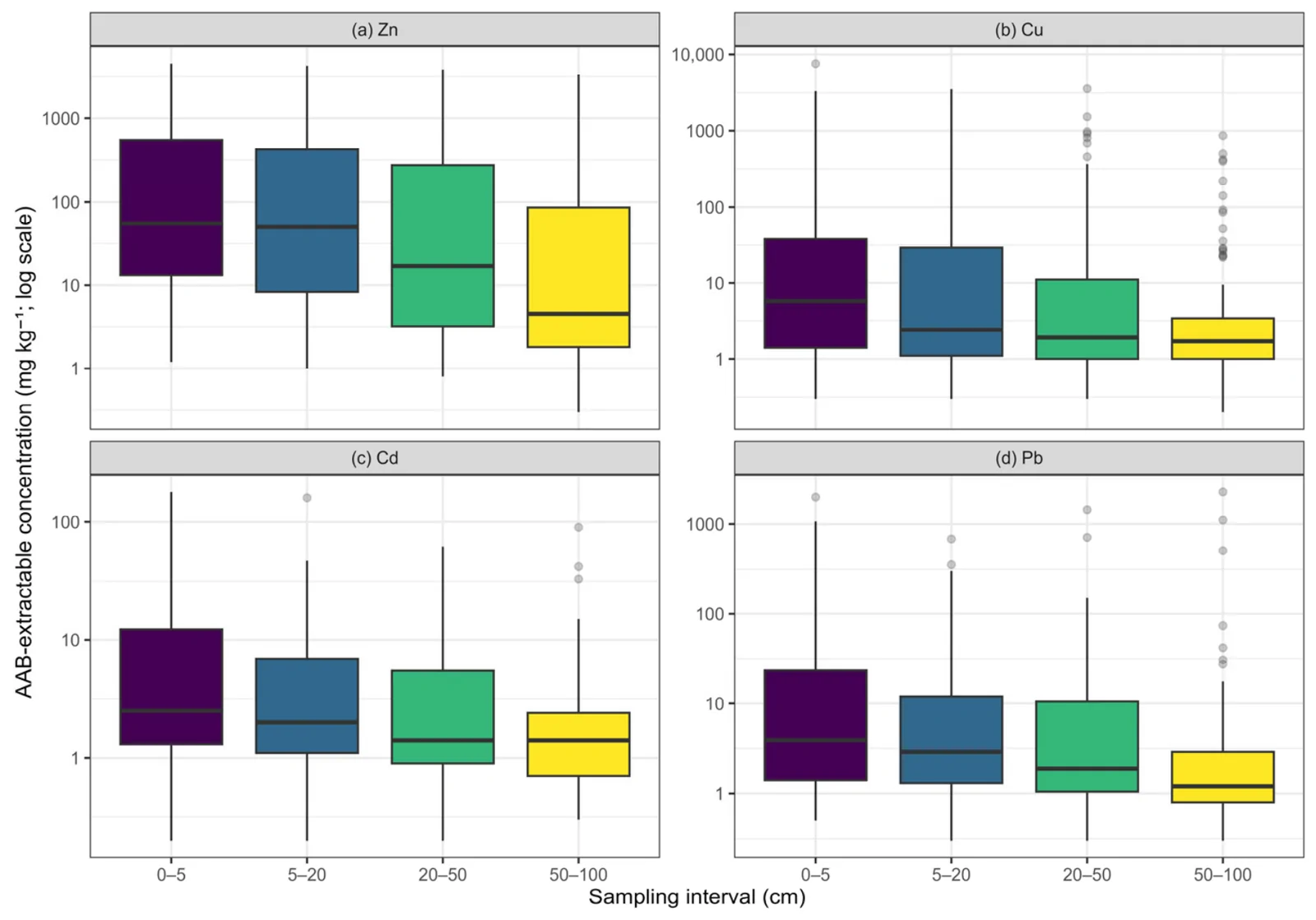
AAB-extractable metal concentrations by depth.

Exchangeable Zn, Cu, Cd, and Pb showed strongly right-skewed distributions and substantial profile-to-profile heterogeneity. Surface concentrations ranged from 0.2 to 179 mg kg^−1^ for Cd, 0.3 to 7585 mg kg^−1^ for Cu, 0.5 to 1996 mg kg^−1^ for Pb, and 1.2 to 4510 mg kg^−1^ for Zn in the 0–5 cm interval (Table 1 and Table S2). Nevertheless, median concentrations declined consistently with depth. Median Zn decreased from 55.2 mg kg^−1^ at 0–5 cm to 50.0, 17.0, and 4.5 mg kg^−1^ at 5–20, 20–50 and 50–100 cm, respectively. Differences in Zn concentrations between surface and subsurface layers may reflect vertical transport processes, which can be influenced by the input concentration and water flux through soil [40]. Median Cu declined from 5.7 to 2.4, 1.9 and 1.7 mg kg^−1^. Median Cd declined from 2.5 to 2.0, 1.4, and 1.4 mg kg^−1^, and median Pb declined from 3.9 to 2.9, 1.9, and 1.2 mg kg^−1^. The strongest surface-to-deep median contrast occurred for Zn, whose median at 50–100 cm was about twelve-fold lower than at 0–5 cm. These element-specific differences are expected because Zn, Cu, Cd, and Pb differ in their interactions with soil organic matter, mineral surfaces, carbonates, and exchange sites, and the resulting extractability is further influenced by soil pH, texture, and organic carbon content [41,42,43,44]. Figure 4 further illustrates the overall decline in AAB-extractable metal concentrations with increasing depth for all four metals.

The depth-related trend was most pronounced for Zn and Pb, which showed the largest Kruskal–Wallis test statistics. Similar depth-dependent variability in metal concentrations has been reported in other soil-profile studies, although its direction and magnitude vary among elements and soil settings [10,14,45,46]. Kruskal–Wallis tests confirmed significant differences among depth intervals for Zn (χ^2^ = 44.32, *p* = 1.29 × 10^−9^), Pb (χ^2^ = 33.22, *p* = 2.89 × 10^−7^), Cd (χ^2^ = 25.29, *p* = 1.34 × 10^−5^) and Cu (χ^2^ = 15.33, *p* = 0.0015). Holm-adjusted Dunn tests showed significantly higher concentrations in the 0–5 cm layer than in the 20–50 and 50–100 cm layers for all metals. Concentrations in the 0–5 and 5–20 cm layers did not differ significantly after adjustment. Cadmium and copper each showed significant differences in three depth-pair comparisons. Comparable depth-dependent patterns in heavy-metal concentrations and bioavailability have been reported in other soil-profile studies, although the magnitude and direction of vertical trends vary with metal type, soil properties, land use, and local environmental conditions [47,48]. In contrast, no statistically significant differences were observed between the 0–5 and 5–20 cm intervals for any metal after Holm adjustment (Table 2).

### 3.3. Depth-Only Mixed-Effects Models

Depth-only mixed-effects models showed significant negative associations between soil depth and log-transformed AAB-extractable concentrations of all four metals (Table 3). The estimated depth coefficients were −0.00970 for Zn, −0.00565 for Cu, −0.00420 for Cd, and −0.00513 for Pb. Thus, all metals exhibited a statistically supported decline with increasing depth, with the steepest depth gradient observed for Zn.

Because the responses were modelled on the log scale, the coefficients describe proportional rather than absolute changes. Holding the profile-specific random intercept constant, a 1 cm increase in depth was associated with model-based decreases of approximately 2.2% for Zn, 1.3% for Cu, 1% for Cd, and 1.2% for Pb.

Despite the strong statistical evidence for negative depth gradients, depth alone accounted for only a modest proportion of total variance (marginal R^2^ = 0.024–0.062). In contrast, conditional R^2^ values ranged from 0.749 to 0.864, showing that the full models, including the profile-level random effect, accounted for substantially more variation. This discrepancy highlights substantial variation among soil profiles. Depth captures a consistent mean vertical trend, while differences among profiles remain the dominant source of variation around that trend.

### 3.4. Same-Sample Correlation

**Figure 5:**
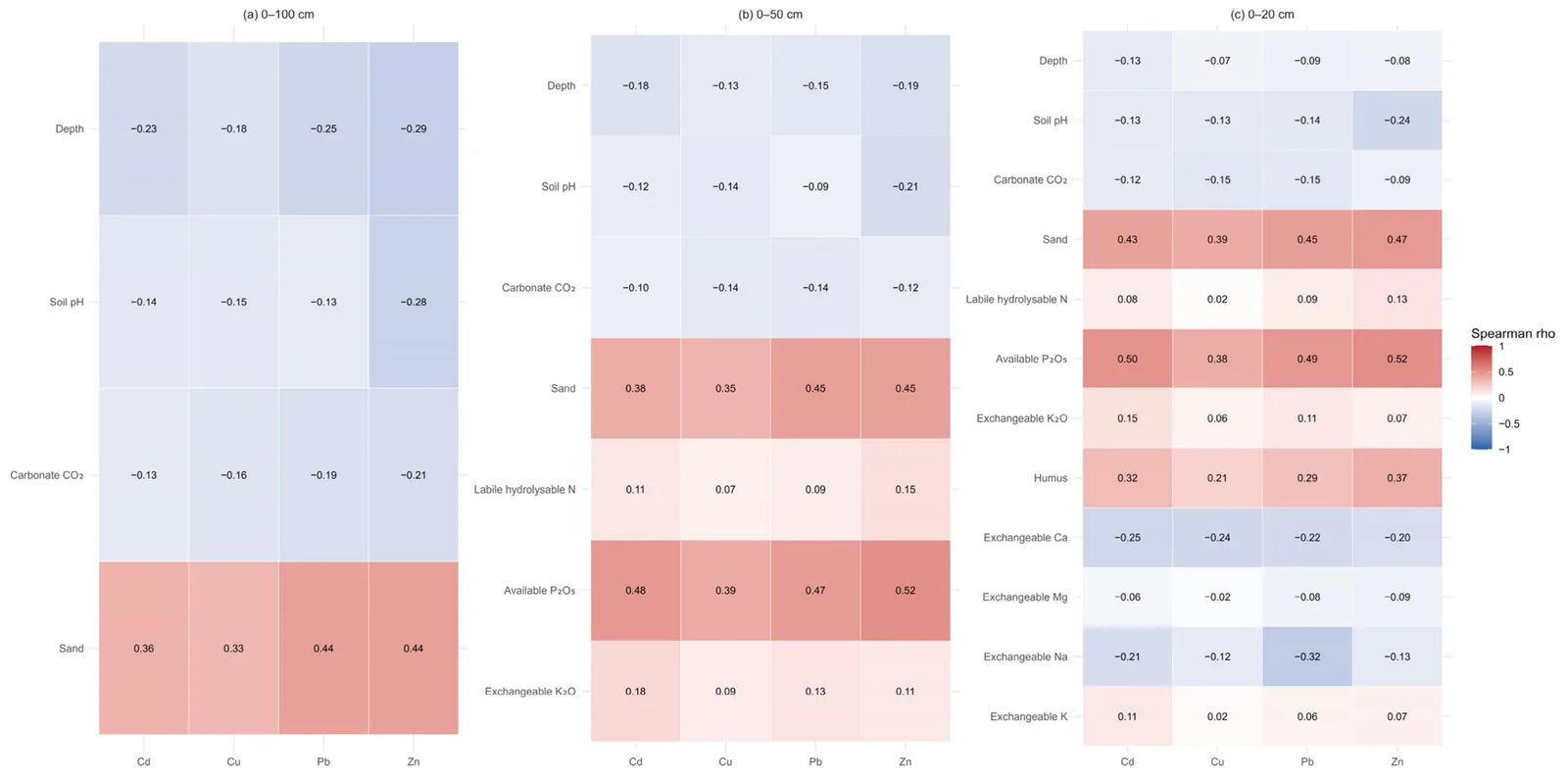
Correlations between metals and soil properties.

Three depth scopes were analysed using progressively more complete sets of soil variables: 0–100 cm, 0–50 cm, and 0–20 cm. Spearman correlations were calculated within each depth scope using the corresponding common complete-case dataset; consequently, the numbers of observations were 486, 393, and 266, respectively. In the 0–100 cm scope, AAB-extractable Zn, Cu, Cd, and Pb were negatively correlated with depth, soil pH, and carbonate CO_2_, whereas all four metals showed positive correlations with sand content (Figure 5a). The strongest depth association was observed for Zn (*ρ* = −0.29) followed by Pb (*ρ* = −0.25), Cd (*ρ* = −0.23), and Cu (*ρ* = −0.18). Zn also showed the strongest negative correlations with pH (*ρ* = −0.28) and carbonate CO_2_ (*ρ* = −0.21). In contrast, sand content was positively associated with all four metals, with the strongest associations observed for Zn (*ρ* = 0.44) and Pb (*ρ* = 0.44), followed by Cd (*ρ* = 0.36) and Cu (*ρ* = 0.33).

In the 0–50 cm scope, available P_2_O_5_ showed the strongest positive associations with all metals (Figure 5b): *ρ* = 0.52 for Zn, *ρ* = 0.48 for Cd, *ρ* = 0.47 for Pb, and *ρ* = 0.39 for Cu. Sand content also remained positively associated with Zn, Cu, Cd, and Pb (*ρ* = 0.35–0.45). Positive associations with labile hydrolysable nitrogen were weaker but remained statistically significant for Zn (*ρ* = 0.15) and Cd (*ρ* = 0.11), whereas exchangeable K_2_O showed weak positive correlations. Within the 0–20 cm scope, the pattern for available P_2_O_5_ remained consistent, with positive correlations for Zn (*ρ* = 0.49), Cu (*ρ* = 0.35), Cd (*ρ* = 0.48) and Pb (*ρ* = 0.49) (Figure 5c). Sand content was again positively correlated with all metals, with coefficients ranging from *ρ* = 0.39 for Cu to *ρ* = 0.47 for Zn. Humus content was also positively associated with all four metals; the strongest associations were observed for Zn (*ρ* = 0.36) and Cd (*ρ* = 0.30), followed by Pb (*ρ* = 0.29) and Cu (*ρ* = 0.19). The cation-inclusive 0–20 cm analysis was based on 264 observations from 132 profiles. Exchangeable Na showed contrasting associations among metals: its strongest negative association was observed for Pb (*ρ* = −0.32), followed by Cd (*ρ* = −0.21), the association with Cu was weaker (*ρ* = −0.12), and that with Zn did not remain significant after Benjamini–Hochberg adjustment (*ρ* = −0.13, *p* = 0.059), exchangeable Ca was negatively associated with Zn (*ρ* = −0.20), Cu (*ρ* = −0.24), Cd (*ρ* = −0.25), and Pb (*ρ* = −0.22). By contrast, Mg and exchangeable K were not significantly associated with the metal concentrations after multiple-testing correction.

Sand content emerged as the most consistently supported particle-size indicator of the AAB-extractable metal pattern. Across the 0–100, 0–50, and 0–20 cm datasets, sand was positively correlated with all four metals (*ρ* = 0.33–0.47). These associations persisted for depth and the soil properties available within each depth scope: the sand coefficients were positive for Zn, Cu, Cd, and Pb in all three extended mixed-effects models. The selected sand fraction should be interpreted as an indicator of the observed soil-texture gradient, not as evidence that sand grains directly retain more metals than finer particles.

This distinction is particularly important when comparing operationally extractable metal concentrations across soils with different physicochemical properties. Experimental studies of Chernozems and Kastanozems have shown that the amounts of loosely bound metals recovered from soil vary with soil properties and extraction conditions [19,20]. Work on Pb adsorption by Chernozem further illustrates the potential involvement of exchangeable cations in metal–soil interactions [49]. Related studies from Kazakhstan provide regional context for the use of acetate-ammonium buffer to assess mobile metal forms [50]. These studies help frame the observed sand and Ca associations.

### 3.5. Same-Sample Mixed-Effects Models

This distinction is important for monitoring design [51,52]. Surface sampling can be informative for identifying the general pattern of elevated AAB-extractable metals in upper horizons, but a single surface measurement or a profile-averaged value may not adequately characterize local conditions.

**Figure 6:**
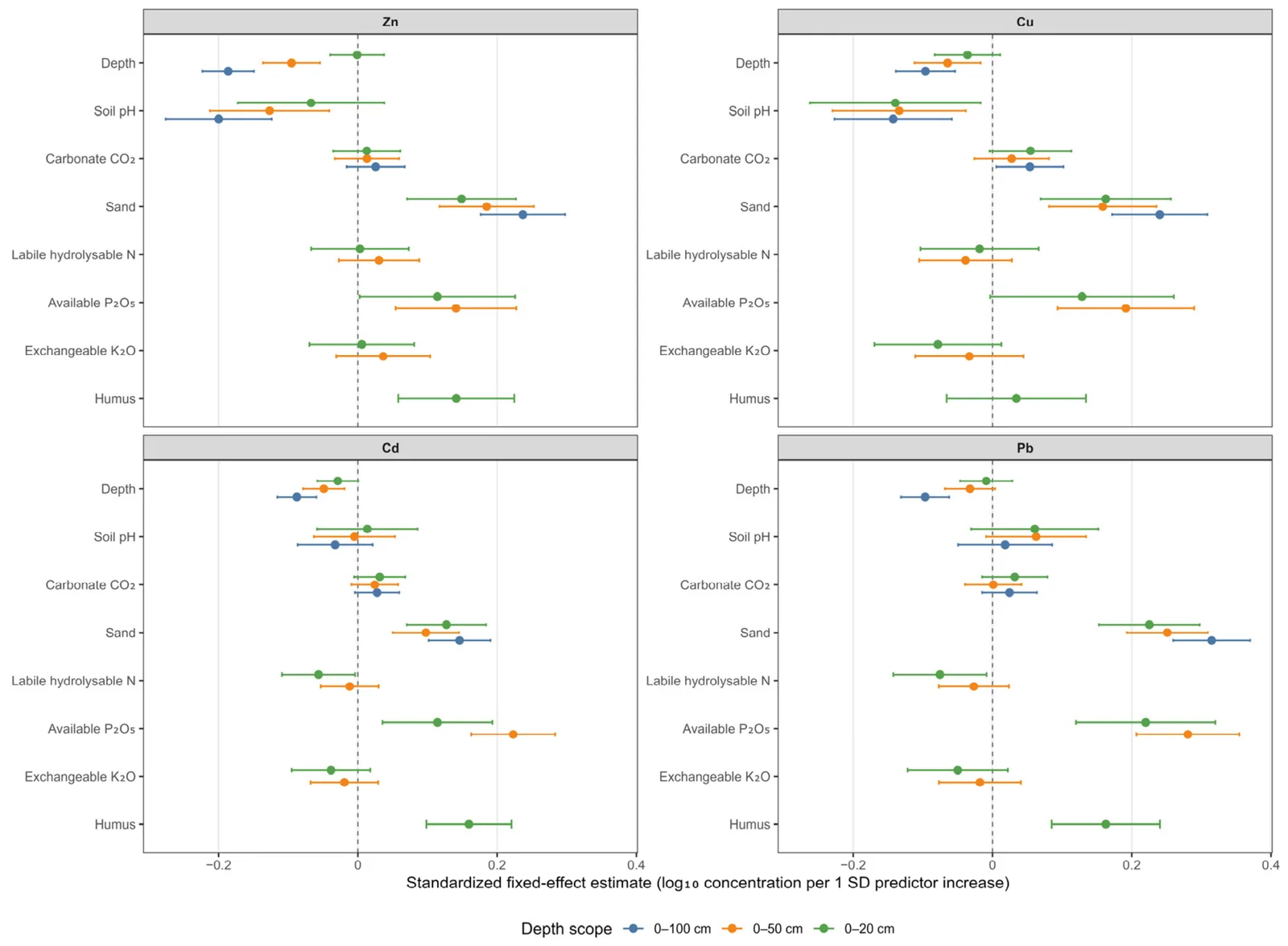
Standardized fixed-effect estimates for soil properties and depth as predictors of AAB-extractable Zn, Cu, Cd, and Pb concentrations across three depth scopes (0–100, 0–50, and 0–20 cm). Note: Points represent coefficient estimates, and horizontal bars indicate uncertainty intervals. The dashed vertical line denotes zero effect.

In depth-only mixed-effects models, concentrations of all four metals decreased with depth (all *p* < 0.001). In the 0–100 cm models, sand content was positively associated with Zn, Cu, Cd and Pb, whereas depth remained negatively associated with all four metals. In the 0–50 cm models, sand and available P_2_O_5_ showed positive associations with every metal. In the 0–20 cm models, sand remained positively associated with all four metals (all *p* < 0.001). Humus content was positively associated with Zn (*β* = 0.048, *p* < 0.001), Cd (*β* = 0.054, *p* < 0.001), and Pb (*β* = 0.055, *p* < 0.001), but not with Cu (*β* = 0.011, *p* < 0.497). Available P_2_O_5_ remained positively associated with Zn (*p* = 0.044), Cd (*p* = 0.004), and Pb (*p* < 0.001), while its association with Cu did not meet the 0.05 threshold (*p* = 0.053) (Figure 6).

### 3.6. Multivariate Patterns

**Figure 7:**
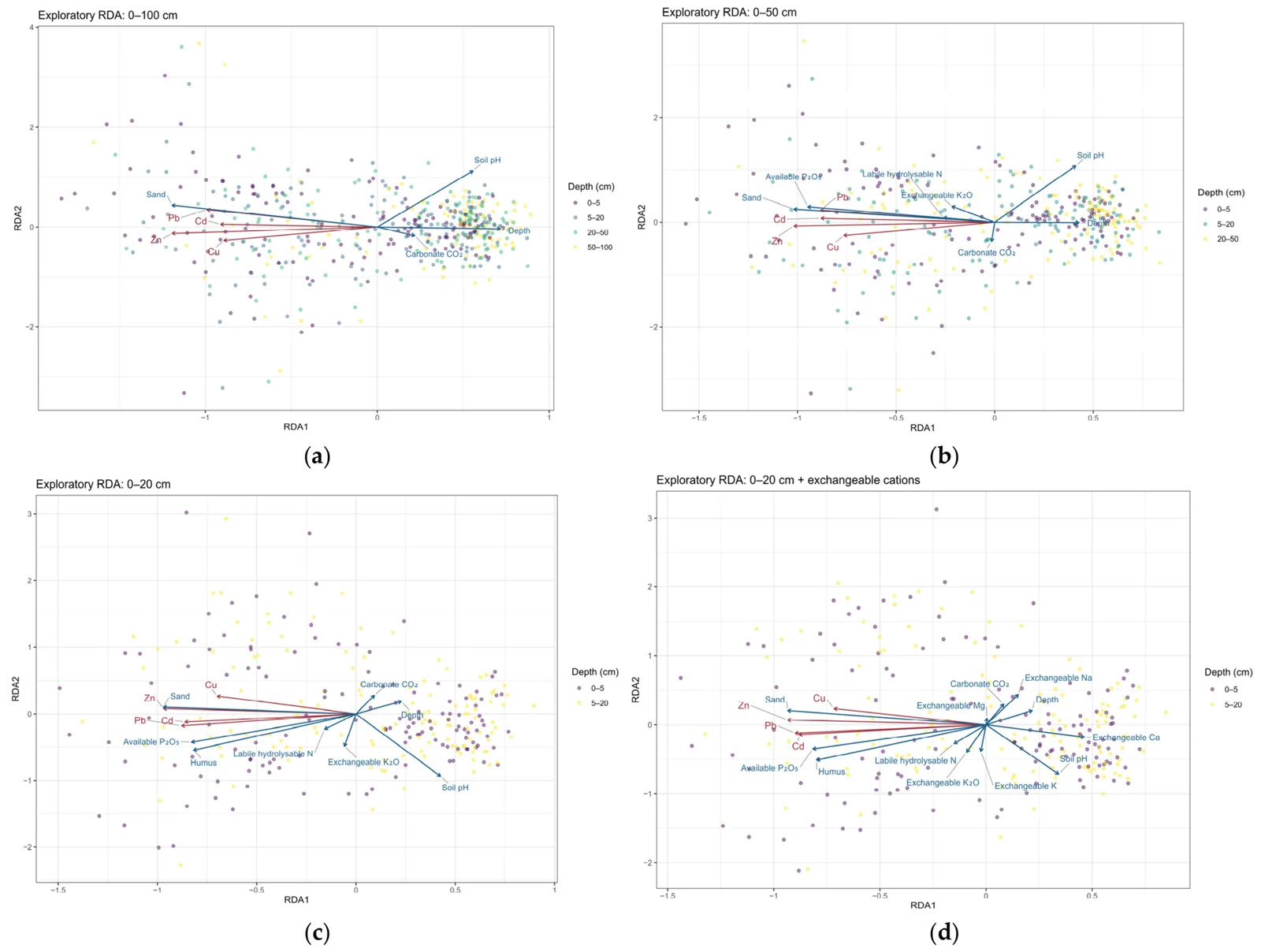
RDA of AAB-extractable metals and soil properties 0–100 cm (**a**), 0–50 cm (**b**), 0–20 cm (**c**), and 0–20 cm with exchangeable cations (**d**).

Exploratory RDA showed increasing constrained separation of the multimetal pattern as the analysis was restricted to shallower soil layers. RDA1 explained 18.4%, 26.3%, and 31.3% of the total variation in standardised log_10_-transformed Zn, Cu, Cd and Pb concentrations in the 0–100, 0–50, and 0–20 (Figure 7) cm scopes, respectively. In all three scopes, sand was the strongest texture-related gradient on RDA1 and was oriented in the same direction as the metal vectors, whereas depth and pH were oriented in the opposite direction. In the 0–50 and 0–20 cm scopes, available P_2_O_5_ was also aligned with the metal vectors. Within 0–20 cm, humus was similarly aligned with the metal gradient, consistent with its positive associations with Zn, Cd and Pb in the mixed-effects models. For the cation-inclusive 0–20 cm analysis (Figure 7d), the multimetal vectors remained aligned with sand, available P_2_O_5_, and humus, whereas exchangeable Ca and soil pH were generally oriented in the opposite direction. Exchangeable Na showed a distinct gradient, consistent with its negative association with Pb and Cd in the correlation analysis. These ordination patterns are exploratory and describe multivariate covariation rather than independent causal effects.

Comparable multivariate patterns have been reported in other studies of metal-affected soils, where the availability of heavy metals was associated with a combination of soil properties rather than with a single environmental variable [27]. For example, an RDA-based study of soils surrounding electroplating facilities showed that pH, silt content and Mn and amorphous Fe oxides were important predictors of the potentially available metal fraction [53]. More broadly, multivariate analyses of contaminated agricultural soils have shown that pH, organic matter, cation-exchange capacity, and texture can differentially influence the available concentrations of Cd, Cu, Pb, Zn, and other metals across contrasting industrial settings [54].

### 3.7. Hotspots and Screening Relevance

**Figure 8:**
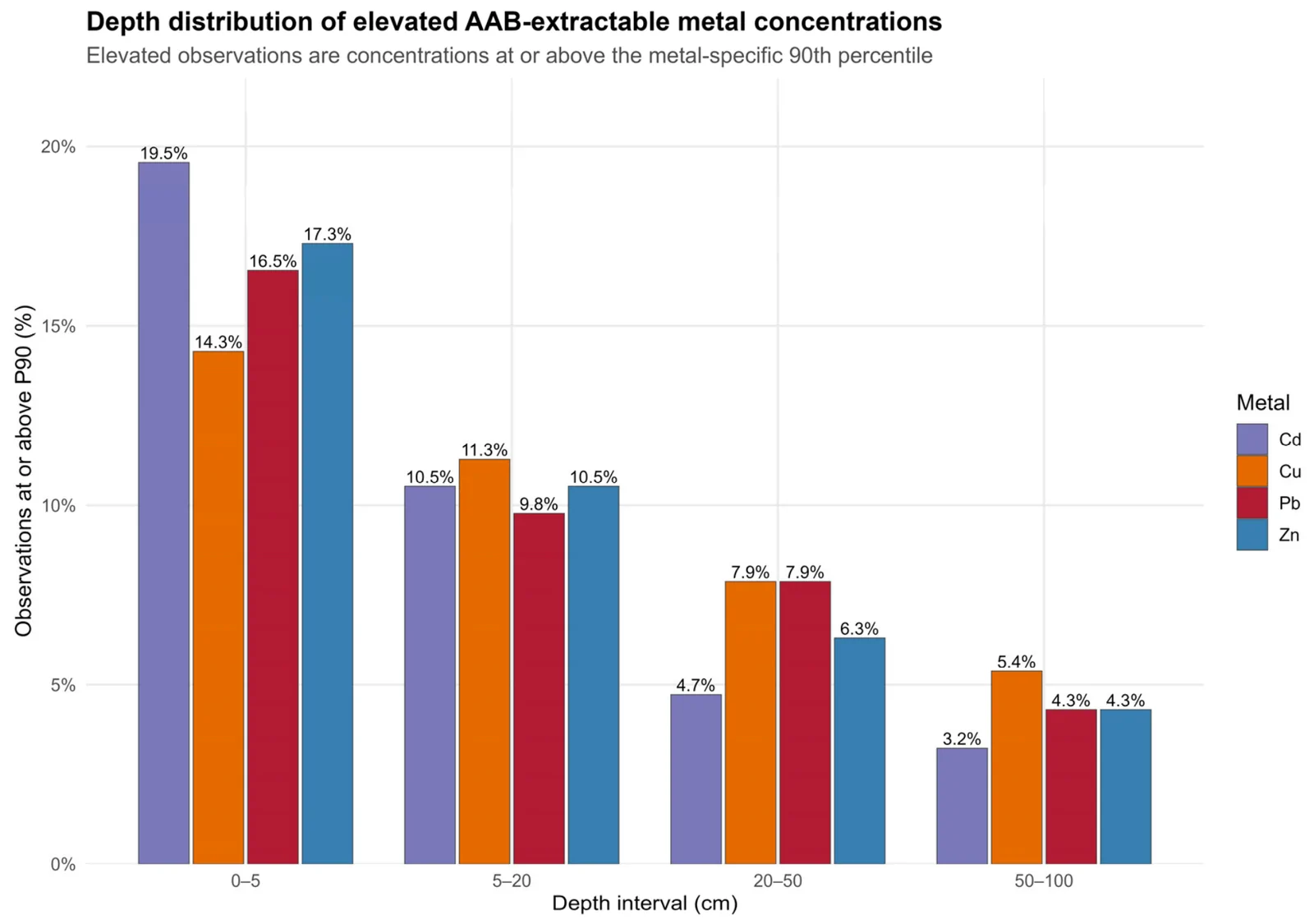
P90-level observations by soil depth.

Metal-specific P90 and P95 thresholds were used as distribution-based indicators of elevated and very elevated observations, respectively. The P90 thresholds were 1074 mg kg^−1^ for Zn, 205.5 mg kg^−1^ for Cu, 17.3 mg kg^−1^ for Cd, and 43.85 mg kg^−1^ for Pb. The frequency of concentrations at or above P90 decreased consistently from the surface to the deepest interval for every metal. In the 0–5 cm interval, 19.5% of Cd observations, 14.3% of Cu observations, 16.5% of Pb observations, and 17.3% of Zn observations met or exceeded their respective P90 thresholds. At 50–100 cm, the corresponding proportions were 3.2% for Cd, 5.4% for Cu, 4.3% for Pb, and 4.3% for Zn (Figure 8). Thus, the most elevated relative concentrations were concentrated mainly in shallow soil, while isolated high-concentration observations were retained at depth.

**Figure 9:**
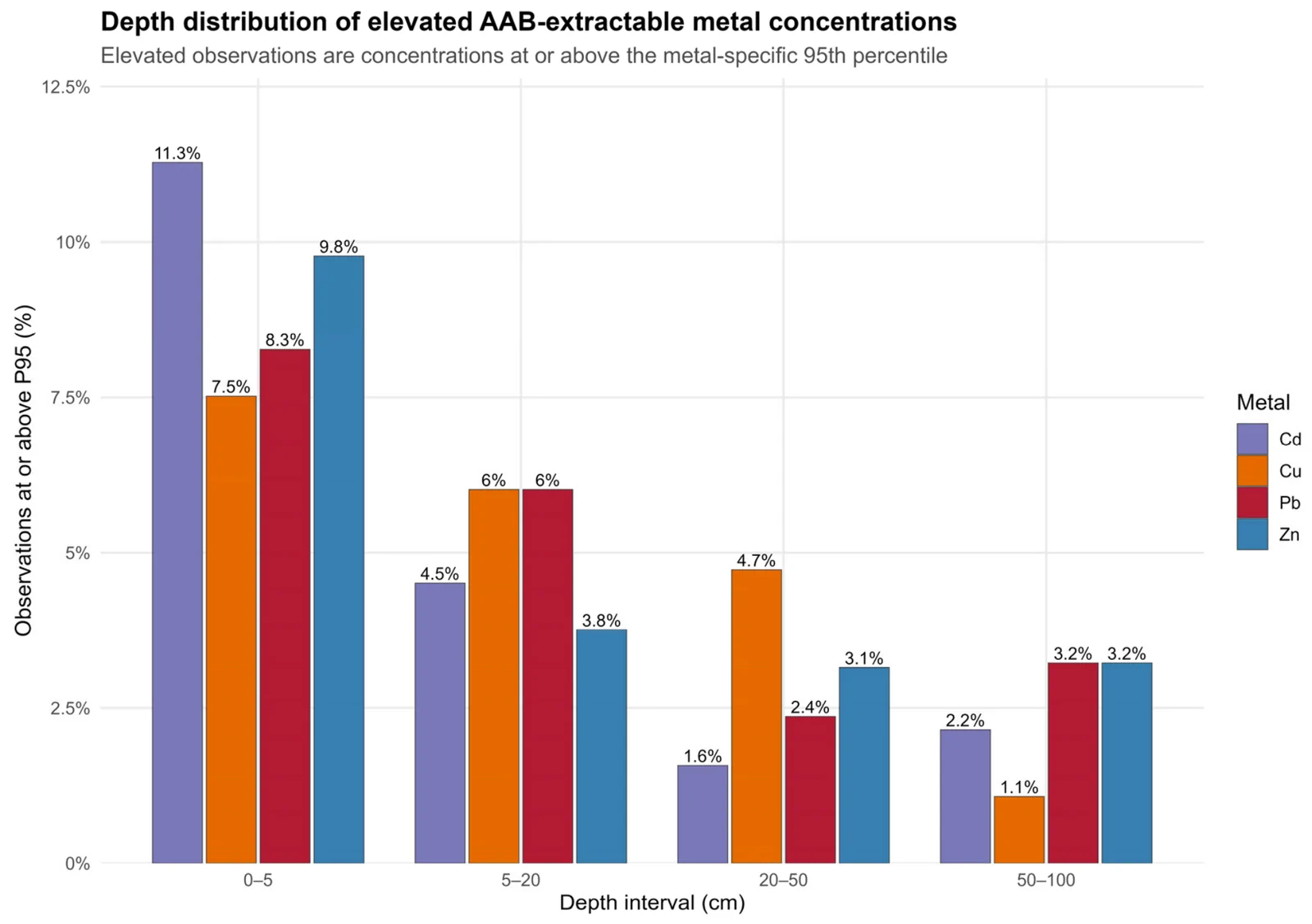
P95-level observations by soil depth.

The P95 results support the same interpretation. Very elevated observations were most frequent in 0–5 cm: 9.8% for Zn, 7.5% for Cu, 11.3% for Cd, and 8.3% for Pb (Figure 9). However, P95-level concentrations still occurred at 50–100 cm for each metal, accounting for 3.2% of Zn observations, 1.1% of Cu observations, 2.2% of Cd observations, and 3.2% of Pb observations. Such observations are consistent with the strong profile-level heterogeneity identified by the mixed models and caution against interpreting the declining median pattern as uniform across all profiles.

The focus on Zn, Cu, Cd, and Pb is relevant to the region’s polymetallic mining and non-ferrous metallurgical setting; previous work in East Kazakhstan has also examined these four elements in metal-affected soils [55]. Their differing depth patterns in the present dataset support considering each AAB-extractable metal separately when prioritising follow-up monitoring, rather than treating the four elements as a single contamination measure.

### 3.8. Study Limitations and Implications for Interpretation

This study has several limitations. First, the observed associations between AAB-extractable metals and soil properties should be interpreted as statistical relationships rather than direct evidence of controlling processes.

Second, the upper-percentile thresholds were calculated from the pooled study dataset and serve only as relative, within-study indicators of elevated observations. They are not regulatory exceedance criteria, toxicological thresholds, or site-specific background values. Because the data were pooled across depth intervals, the screening thresholds primarily identify observations that are high relative to the complete regional dataset; depth-stratified percentile thresholds could be considered in future work where the aim is to identify anomalies separately within individual horizons.

Third, the present study is limited to AAB-extractable Zn, Cu, Cd, and Pb and therefore characterizes an operationally defined extractable pool rather than the total metal content of the soil. Future studies could include total or pseudo-total metal measurements to place this pool in the context of the broader soil metal inventory.

## 4. Conclusions

Extractable Zn, Cu, Cd, and Pb generally decreased with increasing soil depth, with the clearest pattern observed for Zn. Statistically significant differences among depth intervals and negative depth coefficients in the mixed-effects models supported the hypothesis of an overall decline in AAB-extractable metal concentrations with depth. Relatively high concentrations occurred most frequently in the 0–5 cm interval; however, pooled P95 exceedances at 50–100 cm for all metals showed that elevated concentrations were not restricted to surface soil. Sand content was consistently positively associated with all four metals across the analysed depth scopes. Available P_2_O_5_ was positively associated with all four metals in the 0–50 cm models, whereas humus was positively associated with Zn, Cd, and Pb in the upper 20 cm. Within the cation-inclusive 0–20 cm analysis, exchangeable Ca showed negative associations with Cu, Cd and Pb, while Mg was not significantly associated with any metal. The multivariable models and exploratory RDA further showed that depth-dependent changes in physicochemical properties were associated with multimetal variation, although these observational associations do not establish causality. Depth-resolved sampling should therefore be considered where subsurface enrichment is plausible, particularly in heterogeneous polymetallic mining and metallurgical landscapes. The results provide a baseline for future investigations of depth-dependent extractable PTE distributions in the East Kazakhstan Region and can support the design of site-specific soil-monitoring programmes.

## Figures and Tables

**Table 1:** Descriptive statistics for AAB-extractable Zn, Cu, Cd, and Pb concentrations by soil-depth interval.

Depth	Metal	*n*	Mean, mg kg^−1^	SD, mg kg^−1^	Median, mg kg^−1^	MAD, mg kg^−1^	IQR, mg kg^−1^	Min, mg kg^−1^	Max, mg kg^−1^	CV, %	Geometric Mean, mg kg^−1^
0–5	Cd	133	12.2	23.4	2.5	1.6	11.0	0.2	179	191.9	3.9
0–5	Cu	133	193.4	778.5	5.7	4.9	36.4	0.3	7585	402.6	8.8
0–5	Pb	133	60.0	222.2	3.9	2.9	21.9	0.5	1996	370.4	6.3
0–5	Zn	133	510.3	891.3	55.2	52.7	538.8	1.2	4510	174.6	82.7
5–20	Cd	133	7.3	16.1	2.0	1.3	5.8	0.2	160	222.6	2.7
5–20	Cu	133	122.5	425.8	2.4	1.8	28.0	0.3	3516	347.6	6.5
5–20	Pb	133	25.1	78.9	2.9	2.1	10.7	0.3	684	314.7	4.3
5–20	Zn	133	364.6	691.0	50.0	47.1	415.7	1	4245	189.5	58.5
20–50	Cd	127	4.6	8.1	1.4	0.8	4.6	0.2	62	176.0	2.1
20–50	Cu	127	88.1	373.3	1.9	1.1	10.1	0.3	3569	423.5	4.3
20–50	Pb	127	27.8	143.6	1.9	1.2	9.5	0.3	1450	516.5	3.3
20–50	Zn	127	251.8	518.2	17.0	15.8	271.8	0.8	3834	205.8	29.0
50–100	Cd	93	3.9	10.7	1.4	0.7	1.7	0.3	90	272.2	1.6
50–100	Cu	93	33.3	119.6	1.7	0.8	2.4	0.2	865	359.6	2.8
50–100	Pb	93	45.9	265.3	1.2	0.6	2.1	0.3	2276	577.6	2.0
50–100	Zn	93	192.5	483.6	4.5	3.4	84.2	0.3	3334	251.2	12.7

**Table 2:** Depth-related differences in AAB-extractable metals.

Metal	Group1	Group2	n1	n2	Dunn Z	Holm-Adjusted *p*	Significance
Cd	0–5	5–20	133	133	−2.050	0.121	ns
Cd	0–5	20–50	133	127	−3.480	0.003	**
Cd	0–5	50–100	133	93	−4.742	0.000	****
Cd	5–20	20–50	133	127	−1.454	0.251	ns
Cd	5–20	50–100	133	93	−2.882	0.016	*
Cd	20–50	50–100	127	93	−1.533	0.251	ns
Cu	0–5	5–20	133	133	−1.202	0.535	ns
Cu	0–5	20–50	133	127	−2.534	0.050	**
Cu	0–5	50–100	133	93	−3.667	0.001	***
Cu	5–20	20–50	133	127	−1.346	0.535	ns
Cu	5–20	50–100	133	93	−2.576	0.050	**
Cu	20–50	50–100	127	93	−1.328	0.535	ns
Pb	0–5	5–20	133	133	−1.423	0.233	ns
Pb	0–5	20–50	133	127	−2.975	0.012	**
Pb	0–5	50–100	133	93	−5.530	0.000	****
Pb	5–20	20–50	133	127	−1.569	0.233	ns
Pb	5–20	50–100	133	93	−4.239	0.001	***
Pb	20–50	50–100	127	93	−2.772	0.017	*
Zn	0–5	5–20	133	133	−1.217	0.224	ns
Zn	0–5	20–50	133	127	−3.775	0.001	***
Zn	0–5	50–100	133	93	−6.128	0.000	****
Zn	5–20	20–50	133	127	−2.572	0.025	*
Zn	5–20	50–100	133	93	−5.024	0.000	****
Zn	20–50	50–100	127	93	−2.638	0.025	*

Note: Holm-adjusted *p*-values are shown. ns—not significant; *p*_adj_ ≥ 0.05; * *p*_adj_ < 0.05; ** *p*_adj_ < 0.01; *** *p*_adj_ < 0.001; **** *p*_adj_ < 0.0001.

**Table 3:** Depth effects on metal concentrations estimated by linear mixed-effects models.

Metal	Depth Slope, per cm	95% CI	*p*-Value	Marginal *R*^2^	Conditional *R*^2^
Zn	−0.00970	−0.01103 to −0.00837	<0.001	0.06	0.86
Cu	−0.00565	−0.00717 to −0.00414	<0.001	0.02	0.80
Cd	−0.00420	−0.00519 to −0.00321	<0.001	0.04	0.76
Pb	−0.00513	−0.00647 to −0.00380	<0.001	0.03	0.75

## Data Availability

The original contributions presented in this study are included in the article/Supplementary Material. Further inquiries can be directed to the corresponding author.

## Supplementary Materials

The following supporting information can be downloaded at https://www.mdpi.com/article/10.3390/toxics14100918/s1, Table S1: Descriptive statistics of soil physicochemical properties by depth layer; Table S2. Coordinates, sampling depth intervals and AAB-extractable Zn, Cu, Cd and Pb concentrations of the analysed soil samples.
