# Supplementary material for: Extractable Potentially Toxic Elements Across Soil Profiles in East Kazakhstan: Depth Patterns and Soil-Property Associations
Source: Toxics. 2026 Oct 16;14(10):918. doi: 10.3390/toxics14100918 (PMC13709398; doi:10.3390/toxics14100918)
Supplement: Supplementary file 1 [file toxics-14-00918-s001.zip › toxics-4589921-supplementary.pdf]

Supplementary Materials

Article

Depth-Resolved Patterns of Potentially Toxic Elements in Soil Profiles: Associations with Soil Properties and Implications for Environmental Risk Screening

Table S1. Descriptive statistics of soil physicochemical properties by depth layer.

Variable	Depth, cm	n	Median (IQR) [min–max]
Exchangeable Ca, meq 100 g^{-1}	0–5	132	16.83 (8.49) [2.88–32.98]
	5–20	132	17.32 (8.47) [1.92–51.89]
	20–50	0	—
	50–100	0	—
CO_2 value	0–5	133	0.37 (0.32) [0.05–5.04]
	5–20	133	0.37 (0.34) [0.05–5.87]
	20–50	127	0.39 (0.44) [0.05–12.99]
	50–100	93	0.45 (0.87) [0.05–6.16]
Humus, %	0–5	133	4.17 (3.32) [0.72–19.47]
	5–20	133	3.48 (1.97) [0.70–22.74]
	20–50	0	—
	50–100	0	—
Available K_2O, mg kg^{-1}	0–5	133	440 (390) [30–1460]
	5–20	133	370 (240) [20–1080]
	20–50	127	230 (145) [1–1080]
	50–100	0	—
Exchangeable K, meq 100 g^{-1}	0–5	133	0.30 (0.64) [0.01–2.33]
	5–20	133	0.15 (0.26) [0.01–1.99]
	20–50	0	—
	50–100	0	—
Exchangeable Mg, meq 100 g^{-1}	0–5	132	4.36 (2.87) [0.48–15.52]
	5–20	132	4.34 (2.47) [0.97–25.70]
	20–50	0	—
	50–100	0	—
Exchangeable Na, meq 100 g^{-1}	0–5	133	0.12 (0.16) [0.01–0.78]
	5–20	133	0.12 (0.17) [0.01–2.62]
	20–50	0	—
	50–100	0	—
Exchangeable Na share, %	0–5	132	0.61 (0.57) [0.03–5.36]

	5–20	132	0.60 (0.70) [0.03–6.65]
	20–50	0	—
	50–100	0	—
Nitrogen, mg kg^{-1}	0–5	133	58.8 (25.2) [19.6–187.6]
	5–20	133	50.4 (25.2) [19.6–117.6]
	20–50	127	44.8 (22.4) [11.2–142.8]
	50–100	0	—
Available P_2O_5, mg kg^{-1}	0–5	133	50 (76) [6–288]
	5–20	133	30 (30) [4–232]
	20–50	127	16 (26) [2–252]
	50–100	0	—
pH	0–5	133	6.57 (0.87) [3.09–7.48]
	5–20	133	6.65 (1.03) [2.82–7.58]
	20–50	127	6.86 (0.80) [2.66–7.74]
	50–100	93	7.03 (0.88) [2.51–7.60]
Sand (1.0–0.05 mm), %	0–5	133	44.24 (30.08) [2.65–89.92]
	5–20	133	38.86 (33.37) [5.04–91.15]
	20–50	127	34.33 (32.95) [0.92–95.58]
	50–100	93	24.68 (23.86) [2.83–91.57]
Silt (0.05–0.001 mm), %	0–5	133	52.33 (29.22) [9.67–90.74]
	5–20	133	54.58 (34.55) [7.64–92.50]
	20–50	127	61.17 (33.13) [3.21–89.62]
	50–100	93	71.06 (23.05) [7.63–90.76]
Fine fraction (<0.001 mm), %	0–5	133	2.05 (2.86) [0.40–18.04]
	5–20	133	3.24 (4.92) [0.40–30.47]
	20–50	127	4.46 (5.69) [0.40–21.68]
	50–100	93	3.67 (6.11) [0.41–28.04]

Table S2. Coordinates, sampling depth intervals and AAB-extractable Zn, Cu, Cd and Pb concentrations of the analysed soil samples.

Sample No.	Sample ID	Latitude (decimal degrees)	Longitude (decimal degrees)	Sampling depth interval (cm)	AAB-extractable Zn (mg kg^{-1} dry soil)	AAB-extractable Cu (mg kg^{-1} dry soil)	AAB-extractable Cd (mg kg^{-1} dry soil)	AAB-extractable Pb (mg kg^{-1} dry soil)
1	1	50.348908	83.49847	0–5	994	90	9.5	10
2	1	50.348908	83.49847	5–20	186	21.3	1.3	6.4
3	1	50.348908	83.49847	20–50	97	44.6	2.3	1.6
4	1	50.348908	83.49847	50–100	86	51.7	2.4	1.1
5	2	50.35251	83.50465	0–5	3029	375	37	74
6	2	50.35251	83.50465	5–20	1281	96	6.3	12
7	2	50.35251	83.50465	20–50	964	57.3	7.8	15.4
8	2	50.35251	83.50465	50–100	560	3.6	4.6	11.4
9	3	50.341706	83.49398	0–5	552	1.7	5.9	18.2
10	3	50.341706	83.49398	5–20	102	1	1.5	7.9
11	3	50.341706	83.49398	20–50	96	0.8	2.4	8.4
12	4	50.3435	83.48618	0–5	4145	1052	179	405
13	4	50.3435	83.48618	5–20	4245	913	160	243
14	4	50.3435	83.48618	20–50	1784	11.1	10	8.4
15	4	50.3435	83.48618	50–100	1024	6.5	6.2	3.5
16	5	50.34364	83.48352	0–5	3035	189.2	54	80.3
17	5	50.34364	83.48352	5–20	3046	103	47	62
18	5	50.34364	83.48352	20–50	2128	7.4	15.5	11
19	5	50.34364	83.48352	50–100	818	2.6	4.5	3.9
20	6	50.35054	83.48593	0–5	3602.4	325	101	214
21	6	50.35054	83.48593	5–20	3021	31	28	302.6
22	6	50.35054	83.48593	20–50	1817	11.1	9.4	16.5
23	6	50.35054	83.48593	50–100	535	3.4	5	5.9
24	7	50.3489	83.48089	0–5	3235	118	84	947
25	7	50.3489	83.48089	5–20	2008	54	35.9	23.7
26	7	50.3489	83.48089	20–50	537	2.3	5.7	2.6

27	7	50.3489	83.48089	50–100	88	0.3	3	1.1
28	8	50.35185	83.48397	0–5	2928	51	59.2	657.6
29	8	50.35185	83.48397	5–20	1406	41.7	11	17
30	8	50.35185	83.48397	20–50	329	1.9	1.8	3.4
31	8	50.35185	83.48397	50–100	137	0.7	0.7	1.4
32	9	50.36108	83.47661	0–5	2049	220	61	336
33	9	50.36108	83.47661	5–20	1630	19.8	29	22.8
34	9	50.36108	83.47661	20–50	473	5.2	16	7.8
35	9	50.36108	83.47661	50–100	371	21.8	4.8	17.6
36	10	50.3516	83.47301	0–5	1025.2	52	6.9	13.1
37	10	50.3516	83.47301	5–20	376	11.2	9	4.9
38	10	50.3516	83.47301	20–50	753.9	35.7	1.4	14.2
39	10	50.3516	83.47301	50–100	745	22.5	0.6	15.2
40	11	50.35801	83.4885	0–5	308	4	3.3	1
41	11	50.35801	83.4885	5–20	158	0.9	0.4	0.8
42	11	50.35801	83.4885	20–50	68	1.2	0.7	0.8
43	11	50.35801	83.4885	50–100	98	1.3	0.6	0.6
44	12	50.33766	83.49274	0–5	621	7.8	8.4	27.7
45	12	50.33766	83.49274	5–20	69	1.1	2	6.2
46	12	50.33766	83.49274	20–50	116	2.6	1.4	15.5
47	13	50.3445	83.50054	0–5	1261	20.1	40	121.2
48	13	50.3445	83.50054	5–20	1537	103.5	31	205.3
49	13	50.3445	83.50054	20–50	522	9.2	5.3	24.3
50	13	50.3445	83.50054	50–100	240	7.6	4.7	27.4
51	14	50.3523	83.51444	0–5	507	10.3	5.1	21.2
52	14	50.3523	83.51444	5–20	286	8.4	3.2	31.7
53	14	50.3523	83.51444	20–50	530	13.3	5.8	53.8
54	15	50.3613	83.50303	0–5	1885	7	44	28.9
55	15	50.3613	83.50303	5–20	1067	4.1	10	8
56	15	50.3613	83.50303	20–50	498	1.9	5	3.6
57	15	50.3613	83.50303	50–100	46	0.4	0.8	0.8
58	16	50.35783	83.50184	0–5	2758	37.3	42.4	75.9

59	16	50.35783	83.50184	5–20	462	12.7	6	7.1
60	16	50.35783	83.50184	20–50	142	6.5	2	6.3
61	16	50.35783	83.50184	50–100	54	2	1.4	4.8
62	17	50.35832	83.52835	0–5	1002	62.1	17.9	150
63	17	50.35832	83.52835	5–20	1059	293.5	33.2	355.1
64	17	50.35832	83.52835	20–50	1286	454	62	712
65	17	50.35832	83.52835	50–100	1774	498	90	1106
66	18	50.36327	83.5361	0–5	33.3	32	25	4.4
67	18	50.36327	83.5361	5–20	17	1.9	0.8	3
68	18	50.36327	83.5361	20–50	454	11.4	7.5	102
69	18	50.36327	83.5361	50–100	496	9.5	8.4	2276
70	19	50.36414	83.5409	0–5	381	0.7	1.7	16.9
71	19	50.36414	83.5409	5–20	3368	5	13.2	684
72	19	50.36414	83.5409	20–50	3834	2.6	13.7	1450
73	19	50.36414	83.5409	50–100	3334	1	7.9	508
74	20	50.36273	83.55308	0–5	1163	29.9	13.2	1996
75	20	50.36273	83.55308	5–20	424	10.7	9	6.3
76	20	50.36273	83.55308	20–50	276	7.4	5.8	38.6
77	20	50.36273	83.55308	50–100	14	1.7	2.6	5.2
78	21	50.35867	83.55804	0–5	48	1.3	4	6.7
79	21	50.35867	83.55804	5–20	15.8	1.1	2.9	4.9
80	21	50.35867	83.55804	20–50	52	0.9	3.2	4.2
81	21	50.35867	83.55804	50–100	74	0.8	2.5	4.5
82	22	50.35328	83.55063	0–5	576	11.5	18	28.7
83	22	50.35328	83.55063	5–20	94	14.9	6.6	10.9
84	22	50.35328	83.55063	20–50	112	17.8	6.3	12.9
85	22	50.35328	83.55063	50–100	156	28.6	8.4	30.6
86	23	50.35502	83.53120	0–5	1146	13.9	62	205
87	23	50.35502	83.53120	5–20	786	62.3	15.5	79.8
88	23	50.35502	83.53120	20–50	758	50.2	10.1	80
89	24	50.34990	83.53474	0–5	414	6.4	12.3	16.4
90	24	50.34990	83.53474	5–20	693	29.1	27.7	37

91	24	50.34990	83.53474	20–50	763	58.2	11.3	35.8
92	24	50.34990	83.53474	50–100	892	86	7.9	41.9
93	25	50.32597	83.49364	0–5	21.4	0.8	0.9	2.3
94	25	50.32597	83.49364	5–20	61	0.6	1.5	1.9
95	25	50.32597	83.49364	20–50	7.4	0.7	1.4	0.9
96	25	50.32597	83.49364	50–100	3.3	0.8	1.2	0.5
97	26	50.28225	83.32789	0–5	24.8	1.2	2	1.1
98	26	50.28225	83.32789	5–20	19	1.7	2.3	1
99	26	50.28225	83.32789	20–50	8	1.7	2	1.4
100	26	50.28225	83.32789	50–100	3.3	1.3	1.4	1
101	27	50.59838	81.88802	0–5	22.3	4.7	1.6	1.8
102	27	50.59838	81.88802	5–20	34.2	4.5	1.4	1.9
103	28	50.59148	81.8821	0–5	9.7	1.4	0.3	0.7
104	28	50.59148	81.8821	5–20	7.7	0.8	0.6	0.3
105	28	50.59148	81.8821	20–50	1.2	1.3	0.5	1.1
106	28	50.59148	81.8821	50–100	1.1	1.2	0.4	1
107	29	50.5899	81.88684	0–5	138	802	1.6	5.3
108	29	50.5899	81.88684	5–20	606	29.4	2	1.3
109	29	50.5899	81.88684	20–50	7.4	0.9	0.9	1.5
110	29	50.5899	81.88684	50–100	3.5	0.8	1.4	1.4
111	30	50.59035	81.88834	0–5	200	88	2.7	7
112	30	50.59035	81.88834	5–20	226	88	2.5	6
113	30	50.59035	81.88834	20–50	4.2	18.9	1.3	2.5
114	30	50.59035	81.88834	50–100	13.7	6.4	0.8	2.1
115	31	50.58818	81.91605	0–5	3.5	1.5	0.5	1.8
116	31	50.58818	81.91605	5–20	1.6	0.8	0.7	2.3
117	31	50.58818	81.91605	20–50	1.5	0.9	0.2	1.9
118	31	50.58818	81.91605	50–100	1.6	0.9	0.5	1.5
119	32	50.59059	81.90456	0–5	29.2	27.8	0.5	3.6
120	32	50.59059	81.90456	5–20	17.5	1	0.4	1.9
121	33	50.58762	81.88615	0–5	4510	2572	36	2.3
122	33	50.58762	81.88615	5–20	1634	1476	22	1.6

123	33	50.58762	81.88615	20–50	990	914	4.2	1.8
124	33	50.58762	81.88615	50–100	1072	396	4.3	1.5
125	34	50.58513	81.88454	0–5	2108	364	12.2	38
126	34	50.58513	81.88454	5–20	386	6.4	1.3	2.5
127	34	50.58513	81.88454	20–50	36	1.7	0.6	1.6
128	35	50.5868	81.87592	0–5	6.6	1.8	1.3	1
129	35	50.5868	81.87592	5–20	9.7	1.4	1.2	0.5
130	35	50.5868	81.87592	20–50	2.8	0.8	1.5	0.5
131	35	50.5868	81.87592	50–100	3	0.7	2	0.7
132	36	50.58472	81.87515	0–5	6	0.7	2.1	0.7
133	36	50.58472	81.87515	5–20	2.3	1	1.2	1.8
134	36	50.58472	81.87515	20–50	2.9	1.5	1.5	1.9
135	36	50.58472	81.87515	50–100	4.1	2.2	1.5	2
136	37	50.56635	81.86597	0–5	4	1.5	0.2	1.7
137	37	50.56635	81.86597	5–20	3.3	1.4	0.2	1.5
138	37	50.56635	81.86597	20–50	3.7	1.6	0.4	1.4
139	37	50.56635	81.86597	50–100	1.9	1.2	0.3	1
140	38	50.55137	81.86475	0–5	5.3	0.9	2.4	1.2
141	38	50.55137	81.86475	5–20	4.2	0.8	1.1	1.4
142	38	50.55137	81.86475	20–50	2	0.8	1.2	1.2
143	39	50.5502	81.84794	0–5	6.1	0.7	1.4	0.8
144	39	50.5502	81.84794	5–20	6.4	1	1.7	1.2
145	39	50.5502	81.84794	20–50	2	0.3	2.2	0.8
146	39	50.5502	81.84794	50–100	2.5	1.2	1.8	0.6
147	40	50.54305	81.854	0–5	59	3.3	2	2
148	40	50.54305	81.854	5–20	17.2	0.5	1.1	1.3
149	40	50.54305	81.854	20–50	3.2	1.4	0.9	1
150	41	50.55732	81.84732	0–5	2.5	6.4	1.1	1.3
151	41	50.55732	81.84732	5–20	20.3	5.2	0.7	1.3
152	41	50.55732	81.84732	20–50	12.2	0.9	0.8	8.2
153	42	50.28223	81.99877	0–5	2	0.7	0.8	0.6
154	42	50.28223	81.99877	5–20	2.2	0.6	0.3	0.5

155	42	50.28223	81.99877	20–50	1.5	0.9	1.4	1.1
156	42	50.28223	81.99877	50–100	1.2	1.1	1.6	1.2
157	43	50.28759	82.00336	0–5	4.9	1.1	1.5	1
158	43	50.28759	82.00336	5–20	2.9	0.7	1.3	0.9
159	43	50.28759	82.00336	20–50	1.8	0.9	1.4	0.8
160	43	50.28759	82.00336	50–100	1.5	1.3	1.6	0.4
161	44	50.2936	82.01333	0–5	28.1	2.2	1.8	2.1
162	44	50.2936	82.01333	5–20	7.8	1.9	1.3	1.6
163	44	50.2936	82.01333	20–50	1	1.6	0.9	0.9
164	44	50.2936	82.01333	50–100	1.2	2.2	0.7	1
165	45	50.2871	82.01221	0–5	2.3	1.5	1	1
166	45	50.2871	82.01221	5–20	1.3	1.6	0.7	1.3
167	45	50.2871	82.01221	20–50	0.8	1.2	0.6	1.1
168	45	50.2871	82.01221	50–100	0.9	1.2	0.3	1.4
169	46	50.28496	82.00816	0–5	3.1	1	1	1.5
170	46	50.28496	82.00816	5–20	2.4	0.9	1.2	0.8
171	46	50.28496	82.00816	20–50	2.1	0.8	1.2	1.5
172	46	50.28496	82.00816	50–100	3.5	0.8	1.4	1.7
173	47	50.28201	82.00536	0–5	6.1	1	1.4	1.5
174	47	50.28201	82.00536	5–20	3.6	1.2	1.5	1.7
175	47	50.28201	82.00536	20–50	2.2	1.1	1.1	1.4
176	47	50.28201	82.00536	50–100	3.3	1.7	1.5	1.7
177	48	50.27451	82.00117	0–5	5.1	1.4	1.2	1.2
178	48	50.27451	82.00117	5–20	7.1	1.7	1.9	1
179	48	50.27451	82.00117	20–50	2.7	1	1.7	0.7
180	48	50.27451	82.00117	50–100	3.6	0.8	1.9	1.1
181	49	50.27761	82.00551	0–5	78	1.4	0.5	1.4
182	49	50.27761	82.00551	5–20	130	1	0.5	1.7
183	49	50.27761	82.00551	20–50	3.8	0.9	1.2	1.2
184	49	50.27761	82.00551	50–100	4.6	1.4	1	1.2
185	50	50.27736	82.01269	0–5	7.6	1.9	0.4	1.4
186	50	50.27736	82.01269	5–20	4.6	2.2	1	0.8

187	50	50.27736	82.01269	20–50	1.5	1.3	1.7	0.9
188	50	50.27736	82.01269	50–100	1	1.9	1.8	1.2
189	51	50.26856	82.01324	0–5	17	1.9	2.2	1.7
190	51	50.26856	82.01324	5–20	1	1.3	1.9	1.8
191	51	50.26856	82.01324	20–50	2.7	1.2	1.4	1.1
192	51	50.26856	82.01324	50–100	3.5	1.1	1.4	1.2
193	52	50.27731	82.02001	0–5	7	0.9	1.6	1.9
194	52	50.27731	82.02001	5–20	6.1	0.9	1.2	1.4
195	52	50.27731	82.02001	20–50	1.9	0.8	1.6	1.4
196	52	50.27731	82.02001	50–100	1.1	0.9	1.5	1.2
197	53	50.28233	82.02006	0–5	1.6	0.9	1.2	1.5
198	53	50.28233	82.02006	5–20	2.5	0.8	0.8	1.5
199	53	50.28233	82.02006	20–50	2.7	1.3	0.8	1.3
200	53	50.28233	82.02006	50–100	4.6	1.8	1.5	1.7
201	54	50.28175	82.02642	0–5	7.2	1.3	1.6	1
202	54	50.28175	82.02642	5–20	3.5	1.2	2	1
203	54	50.28175	82.02642	20–50	2.5	1.2	2	0.9
204	54	50.28175	82.02642	50–100	1.4	0.9	0.8	0.4
205	55	50.51971	81.92542	0–5	1.2	0.7	1	1.3
206	55	50.51971	81.92542	5–20	1.1	0.8	0.9	1.5
207	55	50.51971	81.92542	20–50	1.3	1.1	0.9	0.7
208	55	50.51971	81.92542	50–100	2	0.9	1.3	0.9
209	56	50.49813	81.90027	0–5	5.3	0.4	0.9	0.7
210	56	50.49813	81.90027	5–20	3	1.1	0.6	0.6
211	56	50.49813	81.90027	20–50	3.2	1.7	0.6	0.9
212	56	50.49813	81.90027	50–100	3.6	2.1	0.7	0.8
213	57	50.28636	82.0293	0–5	4.2	2	0.7	1.2
214	57	50.28636	82.0293	5–20	3.1	1.9	0.6	0.7
215	57	50.28636	82.0293	20–50	2.3	2.4	0.7	0.7
216	57	50.28636	82.0293	50–100	2.3	2.9	0.9	0.6
217	58	50.28922	82.01913	0–5	13.3	2.5	1.2	2.5
218	58	50.28922	82.01913	5–20	3.8	2.1	1.1	1.1

219	58	50.28922	82.01913	20–50	1	1.9	1.1	0.5
220	58	50.28922	82.01913	50–100	1.6	1.7	1.4	0.8
221	59	50.27333	82.02718	0–5	1.4	1.4	1.5	0.8
222	59	50.27333	82.02718	5–20	3.1	1.1	1.5	0.7
223	59	50.27333	82.02718	20–50	3	1.4	1.2	1.1
224	59	50.27333	82.02718	50–100	3.8	0.9	1.3	1.2
225	60	50.26299	82.07352	0–5	22.9	2.6	1.1	3.1
226	60	50.26299	82.07352	5–20	6.3	0.9	0.9	1.4
227	60	50.26299	82.07352	20–50	1.6	1.2	0.4	1.1
228	61	50.17215	82.27604	0–5	13.2	4.4	1.1	3.7
229	61	50.17215	82.27604	5–20	11.6	4.1	1	3.7
230	61	50.17215	82.27604	20–50	20.7	6.9	2.5	5.8
231	61	50.17215	82.27604	50–100	5.9	2.4	2.3	3.1
232	62	50.16668	82.27747	0–5	32	11.5	3.5	7.4
233	62	50.16668	82.27747	5–20	50	12.4	4.1	7.4
234	62	50.16668	82.27747	20–50	33	3.2	0.6	2.9
235	62	50.16668	82.27747	50–100	24	0.8	0.4	1.1
236	63	50.16198	82.28194	0–5	44.9	980	5	82
237	63	50.16198	82.28194	5–20	274	134	3.6	14.8
238	63	50.16198	82.28194	20–50	8.1	1.9	0.9	1.1
239	63	50.16198	82.28194	50–100	4.3	2.4	1.3	0.6
240	64	50.15836	82.28342	0–5	209	948	5.4	86
241	64	50.15836	82.28342	5–20	453	387	12.1	12.7
242	64	50.15836	82.28342	20–50	337	693	9.5	46.8
243	65	50.15356	82.29121	0–5	101	393	2.7	23.3
244	65	50.15356	82.29121	5–20	97	608	2.3	53
245	65	50.15356	82.29121	20–50	182.2	117.9	7.8	2.5
246	65	50.15356	82.29121	50–100	2.7	1.5	1.1	1
247	66	50.16114	82.28607	0–5	42.9	37.8	4.1	12.9
248	66	50.16114	82.28607	5–20	43.8	43	4	12.6
249	66	50.16114	82.28607	20–50	39.9	57.6	3.6	15.6
250	66	50.16114	82.28607	50–100	1.6	1	1.9	1.8

251	67	50.16161	82.29649	0–5	17.9	5.7	3	4.3
252	67	50.16161	82.29649	5–20	18.5	6.2	3.4	4
253	67	50.16161	82.29649	20–50	5.8	1.9	2.4	2.5
254	67	50.16161	82.29649	50–100	3	2.1	2	2.2
255	68	50.10217	82.17207	0–5	16.7	6	3.3	4
256	68	50.10217	82.17207	5–20	14.4	5.4	3	3.1
257	68	50.10217	82.17207	20–50	2.6	2.4	2	0.9
258	68	50.10217	82.17207	50–100	1.4	1.9	2.1	1.1
259	69	50.14994	82.30607	0–5	286	277	11.3	47.5
260	69	50.14994	82.30607	5–20	177	248	7.7	26.7
261	69	50.14994	82.30607	20–50	86	119	2.8	12.2
262	70	50.14526	82.31932	0–5	26	37.6	1.7	4.9
263	70	50.14526	82.31932	5–20	18	37	1.2	2.9
264	70	50.14526	82.31932	20–50	15	5.8	0.6	1.5
265	71	50.14382	82.31476	0–5	415	22.7	10.6	33.6
266	71	50.14382	82.31476	5–20	230	192	6.8	23.5
267	71	50.14382	82.31476	20–50	194	152	5.5	18.8
268	71	50.14382	82.31476	50–100	231	220	4.6	17.5
269	72	50.14129	82.31808	0–5	47	42.5	1.8	5.1
270	72	50.14129	82.31808	5–20	4.1	1.7	0.6	1.7
271	72	50.14129	82.31808	20–50	4	1.2	0.7	1.6
272	73	50.14113	82.31418	0–5	253	92	7.1	20.4
273	73	50.14113	82.31418	5–20	378	211	11.3	29.8
274	74	50.13855	82.30999	0–5	439	552	14	42.7
275	74	50.13855	82.30999	5–20	450	219	4.6	11.4
276	74	50.13855	82.30999	20–50	274	260	5.3	13.7
277	74	50.13855	82.30999	50–100	31	27.7	1.6	5
278	75	50.13449	82.30804	0–5	21	11	1.8	3.2
279	75	50.13449	82.30804	5–20	54	47.4	1.7	3.6
280	75	50.13449	82.30804	20–50	74	40.4	1.3	3.1
281	76	50.13501	82.31788	0–5	432	751	18	86
282	76	50.13501	82.31788	5–20	763	1724	23	124

283	76	50.13501	82.31788	20–50	772	1519	19	101
284	76	50.13501	82.31788	50–100	292	417	8.4	4.9
285	77	50.14329	82.30751	0–5	1342	1768	30	145
286	77	50.14329	82.30751	5–20	1034	981	15	81
287	77	50.14329	82.30751	20–50	320	365	8	27.5
288	77	50.14329	82.30751	50–100	77	92	33	6.2
289	78	50.14268	82.30089	0–5	1756	7585	89	1071
290	78	50.14268	82.30089	5–20	797	1784	26	223
291	78	50.14268	82.30089	20–50	717	973	25	123
292	79	50.14866	82.2598	0–5	456	46	3.5	9.1
293	79	50.14866	82.2598	5–20	586	50	3.6	10.1
294	79	50.14866	82.2598	20–50	335	205	6.5	34.5
295	79	50.14866	82.2598	50–100	621	865	15	73.9
296	80	50.13976	82.29564	0–5	485	1141	10	39.6
297	80	50.13976	82.29564	5–20	439	1515	12	22.5
298	80	50.13976	82.29564	20–50	204	814	5.4	12
299	81	50.13959	82.29809	0–5	1010	3314	38	215
300	81	50.13959	82.29809	5–20	1014	3516	37	134
301	81	50.13959	82.29809	20–50	1090	3569	43	151
302	82	50.1329	82.33899	0–5	52	68	2.3	7.2
303	82	50.1329	82.33899	5–20	34	21	2	7.2
304	82	50.1329	82.33899	20–50	17	3.4	0.9	1.7
305	82	50.1329	82.33899	50–100	14	1.8	0.6	1.2
306	83	50.13001	82.51752	0–5	69	1.5	1.4	2
307	83	50.13001	82.51752	5–20	63	1.9	1.5	1.9
308	83	50.13001	82.51752	20–50	4	0.9	0.5	1.1
309	83	50.13001	82.51752	50–100	6.7	0.5	0.7	1
310	84	50.13154	82.51564	0–5	770	247	11	4.1
311	84	50.13154	82.51564	5–20	1144	240	13.7	25.4
312	84	50.13154	82.51564	20–50	567	49.7	5.6	7.7
313	84	50.13154	82.51564	50–100	411	35.5	3.7	11.5
314	85	50.13022	82.51026	0–5	451	8.1	7.1	5.8

315	85	50.13022	82.51026	5–20	426	7.3	5.6	4.5
316	85	50.13022	82.51026	20–50	186	4.1	3.7	2.7
317	85	50.13022	82.51026	50–100	43	1.2	1.3	0.5
318	86	50.12699	82.50821	0–5	1248	314	15.1	6.1
319	86	50.12699	82.50821	5–20	1608	432	18.2	5.8
320	86	50.12699	82.50821	20–50	1626	208	29	10
321	86	50.12699	82.50821	50–100	1885	141	42	9.9
322	87	50.124550	82.505048	0–5	435	91	3.3	2
323	87	50.124550	82.505048	5–20	574	132	4.8	3.3
324	87	50.124550	82.505048	20–50	388	16.9	3.6	3.1
325	87	50.124550	82.505048	50–100	949	26.1	11.9	1
326	88	50.12388	82.51159	0–5	124	3.7	2.7	2.7
327	88	50.12388	82.51159	5–20	146	5.6	2.9	2.8
328	88	50.12388	82.51159	20–50	115	5.9	2.6	1.4
329	88	50.12388	82.51159	50–100	15.1	4.8	2.1	0.8
330	89	50.11812	82.50564	0–5	12.7	0.7	1	0.7
331	89	50.11812	82.50564	5–20	13.1	0.7	0.6	0.4
332	89	50.11812	82.50564	20–50	12.1	0.3	0.7	0.5
333	89	50.11812	82.50564	50–100	5	2.7	0.5	0.4
334	90	50.12207	82.49539	0–5	224	0.9	2.5	1.4
335	90	50.12207	82.49539	5–20	54	2.5	1.1	1.1
336	90	50.12207	82.49539	20–50	45.6	9.3	1.3	1.3
337	90	50.12207	82.49539	50–100	452	1.6	3.6	2.4
338	91	50.11542	82.48657	0–5	6.7	1.7	0.4	0.7
339	91	50.11542	82.48657	5–20	8	1.7	0.6	0.5
340	91	50.11542	82.48657	20–50	1.2	1.5	0.4	0.3
341	91	50.11542	82.48657	50–100	0.5	1.8	0.7	0.7
342	92	50.13001	82.47109	0–5	12.5	2	1.3	0.9
343	92	50.13001	82.47109	5–20	13.4	1.5	1.1	0.9
344	92	50.13001	82.47109	20–50	6.1	1	1.3	1.2
345	92	50.13001	82.47109	50–100	0.8	23.3	0.6	0.8
346	93	50.13008	82.4870	0–5	132	10.6	3.7	4.4

347	93	50.13008	82.4870	5–20	317	0.8	3.6	1.8
348	93	50.13008	82.4870	20–50	6	1.5	0.4	0.9
349	93	50.13008	82.4870	50–100	4.5	1.1	0.5	0.6
350	94	50.13712	82.49331	0–5	26.7	1	0.9	1.1
351	94	50.13712	82.49331	5–20	27.4	0.9	1.1	1
352	94	50.13712	82.49331	20–50	12	1.2	0.5	0.6
353	94	50.13712	82.49331	50–100	2	7.2	0.8	0.8
354	95	50.13491	82.50073	0–5	111	0.9	1.8	1.4
355	95	50.13491	82.50073	5–20	25.4	0.7	0.5	0.7
356	95	50.13491	82.50073	20–50	2	1	0.4	0.8
357	95	50.13491	82.50073	50–100	5.9	1	0.4	0.8
358	96	50.13816	82.50628	0–5	26.2	0.9	1.1	1
359	96	50.13816	82.50628	5–20	25.8	0.6	1.4	1
360	96	50.13816	82.50628	20–50	4.3	0.7	1.2	1.2
361	96	50.13816	82.50628	50–100	0.9	0.4	0.6	0.7
362	97	50.13433	82.5047	0–5	260	46	3.8	4.3
363	97	50.13433	82.5047	5–20	178	10.7	2.8	2.6
364	97	50.13433	82.5047	20–50	6.4	1	1.1	0.7
365	97	50.13433	82.5047	50–100	1.8	1.7	1.3	0.7
366	98	50.08394	82.55981	0–5	3.6	1.6	0.4	1
367	98	50.08394	82.55981	5–20	4	1.5	0.5	1
368	98	50.08394	82.55981	20–50	2.1	1.8	0.7	1.2
369	98	50.08394	82.55981	50–100	1	1.8	0.6	0.9
370	99	50.08388	82.56029	0–5	55.2	2.3	1.8	2.4
371	99	50.08388	82.56029	5–20	32.7	1.7	1.1	1.7
372	99	50.08388	82.56029	20–50	3.8	1.2	0.2	0.7
373	99	50.08388	82.56029	50–100	2	1.2	0.7	0.6
374	100	50.03407	82.73841	0–5	40.3	1.4	2.5	2.1
375	100	50.03407	82.73841	5–20	27	0.8	2.1	1.8
376	100	50.03407	82.73841	20–50	5.9	0.4	1.6	1.1
377	100	50.03407	82.73841	50–100	4.9	0.4	1.7	1
378	101	50.04106	82.74651	0–5	4.1	1.7	1.2	0.5

379	101	50.04106	82.74651	5–20	5.6	1.9	1.2	0.8
380	101	50.04106	82.74651	20–50	6.2	2.6	1.3	0.5
381	101	50.04106	82.74651	50–100	2.4	2.1	1.4	0.6
382	102	50.05145	82.73312	0–5	6	2.2	1.1	0.8
383	102	50.05145	82.73312	5–20	6.7	2.1	0.8	0.5
384	102	50.05145	82.73312	20–50	4	2.2	0.7	0.6
385	102	50.05145	82.73312	50–100	1.8	1.7	0.4	0.7
386	103	50.04492	82.75978	0–5	16.5	0.4	0.9	1.1
387	103	50.04492	82.75978	5–20	5.9	0.3	0.5	0.6
388	103	50.04492	82.75978	20–50	1.9	0.7	0.4	0.8
389	103	50.04492	82.75978	50–100	1.1	0.8	0.5	0.6
390	104	50.03699	82.76094	0–5	24.1	1.3	1.1	1.2
391	104	50.03699	82.76094	5–20	8.3	1.2	0.6	1.1
392	104	50.03699	82.76094	20–50	2.5	1.1	0.8	0.4
393	104	50.03699	82.76094	50–100	1.6	1.4	1	0.9
394	105	50.03641	82.77012	0–5	5.2	1.7	1.4	1.2
395	105	50.03641	82.77012	5–20	17.8	1.3	1.7	1.5
396	105	50.03641	82.77012	20–50	3.4	1	1.3	1
397	105	50.03641	82.77012	50–100	0.3	0.9	1.2	0.8
398	106	50.01948	82.4676	0–5	15.9	1.2	1.6	1.7
399	106	50.01948	82.4676	5–20	3.8	0.9	1.1	1.3
400	106	50.01948	82.4676	20–50	3	1.2	1.4	1.2
401	106	50.01948	82.4676	50–100	2.9	1.7	1.5	1.2
402	107	50.01969	82.47295	0–5	7.9	0.9	1	1.1
403	107	50.01969	82.47295	5–20	2.4	1	0.9	0.9
404	107	50.01969	82.47295	20–50	5.3	1	0.9	0.8
405	107	50.01969	82.47295	50–100	8	1.1	1	1.2
406	108	50.04055	82.78061	0–5	8.8	0.9	0.6	1
407	108	50.04055	82.78061	5–20	5.5	0.7	0.7	0.7
408	108	50.04055	82.78061	20–50	3.2	0.5	0.8	0.4
409	108	50.04055	82.78061	50–100	1.6	0.7	1	0.3
410	109	50.02683	82.77137	0–5	10	0.6	1	0.7

411	109	50.02683	82.77137	5–20	15.6	0.6	1.1	0.7
412	109	50.02683	82.77137	20–50	21.1	0.4	1.2	0.6
413	110	50.02356	82.7699	0–5	39	0.4	1.3	1
414	110	50.02356	82.7699	5–20	31.6	0.4	1.3	0.9
415	110	50.02356	82.7699	20–50	11.6	0.4	0.9	0.5
416	110	50.02356	82.7699	50–100	1.7	0.2	0.7	0.4
417	111	50.02168	82.76043	0–5	48.3	1	1.7	1.6
418	111	50.02168	82.76043	5–20	18.7	2.4	1.2	2.9
419	111	50.02168	82.76043	20–50	2.4	2	1	2.4
420	111	50.02168	82.76043	50–100	0.8	2.4	0.5	1.8
421	112	50.02548	82.75712	0–5	9.7	2.9	3	1.9
422	112	50.02548	82.75712	5–20	9.8	3.3	2.1	1.8
423	112	50.02548	82.75712	20–50	10.8	2.1	2.6	2
424	113	50.02663	82.74877	0–5	39.8	1.7	2.5	1.8
425	113	50.02663	82.74877	5–20	26.1	1.4	2.3	1.6
426	113	50.02663	82.74877	20–50	9.1	0.9	1.4	1.7
427	114	50.02913	82.75326	0–5	22	2.3	1.5	3.9
428	114	50.02913	82.75326	5–20	22.7	4.5	0.9	4.1
429	114	50.02913	82.75326	20–50	9.6	0.8	0.8	3
430	115	50.03315	82.75019	0–5	41.7	0.9	2.2	3.1
431	115	50.03315	82.75019	5–20	36.3	1.3	2.1	3.4
432	116	49.96553	82.61949	0–5	333	11.1	26.7	13.5
433	116	49.96553	82.61949	5–20	299	19.1	17.6	12.1
434	116	49.96553	82.61949	20–50	187	5	16.7	4.5
435	117	49.97635	82.61951	0–5	274	11.9	12.6	21.2
436	117	49.97635	82.61951	5–20	8.9	2.4	7.9	8.5
437	117	49.97635	82.61951	20–50	54.3	2	6.4	7.2
438	118	49.97989	82.63309	0–5	434	16.7	18.5	26.7
439	118	49.97989	82.63309	5–20	236	15.6	10.7	16.6
440	118	49.97989	82.63309	20–50	253	16.8	10.3	17.1
441	119	49.97708	82.64009	0–5	1074	5.7	20.4	54
442	119	49.97708	82.64009	5–20	95	2.2	1.4	5

443	119	49.97708	82.64009	20–50	753	32.6	9.7	34.9
444	120	49.98741	82.63811	0–5	1225	13.4	29.6	41.4
445	120	49.98741	82.63811	5–20	208	3.6	3.6	6.2
446	121	49.98822	82.63276	0–5	1125	9.3	12.3	26.5
447	121	49.98822	82.63276	5–20	944	7.3	7.1	19.7
448	121	49.98822	82.63276	20–50	777	5	8.1	17.2
449	122	49.99636	82.62959	0–5	1675	20.1	31.1	59.5
450	122	49.99636	82.62959	5–20	931	8.7	34.4	23.8
451	122	49.99636	82.62959	20–50	96	0.5	2.7	3.1
452	123	49.99599	82.62042	0–5	1582	35.9	39	2.3
453	123	49.99599	82.62042	5–20	546	28.6	13.2	29.7
454	123	49.99599	82.62042	20–50	289	6.2	6.3	11.6
455	124	50.00139	82.61688	0–5	332	5.8	6.1	16.5
456	124	50.00139	82.61688	5–20	364	6.9	6.9	21.7
457	124	50.00139	82.61688	20–50	246	4.2	4.6	14.6
458	125	50.01009	82.63143	0–5	87	0.6	1.9	4.3
459	125	50.01009	82.63143	5–20	95	1.1	2.3	4.2
460	125	50.01009	82.63143	20–50	40	0.7	1.4	2.1
461	125	50.01009	82.63143	50–100	42	1	1.7	2.6
462	126	50.01319	82.58185	0–5	162	0.6	3.8	5.3
463	126	50.01319	82.58185	5–20	126	1.1	3	4.8
464	126	50.01319	82.58185	20–50	9.5	0.9	1.1	2.3
465	126	50.01319	82.58185	50–100	35	0.8	1.2	2.9
466	127	50.0008	82.58355	0–5	594	6.3	11.5	22.7
467	127	50.0008	82.58355	5–20	110	2.2	1.9	6.1
468	127	50.0008	82.58355	20–50	37.9	0.9	1.2	5
469	127	50.0008	82.58355	50–100	5.7	1.2	0.8	2.9
470	128	49.98980	82.58381	0–5	816	22.7	13.4	43.6
471	128	49.98980	82.58381	5–20	859	38.2	10.8	51.3
472	128	49.98980	82.58381	20–50	605	19.7	7.9	38.2
473	129	49.98526	82.5967	0–5	1147	57.8	25	35.9
474	129	49.98526	82.5967	5–20	690	40	12.1	22.6

475	129	49.98526	82.5967	20–50	1142	74	28	59.2
476	130	49.97575	82.60201	0–5	860	6.3	25	21.2
477	130	49.97575	82.60201	5–20	386	4.8	6	6.3
478	130	49.97575	82.60201	20–50	82	2.8	3	2.4
479	131	49.97844	82.5858	0–5	205	13.9	4.1	5.8
480	131	49.97844	82.5858	5–20	107	8.4	2.6	3.7
481	132	49.95752	82.58084	0–5	54	6.9	2.4	2.2
482	132	49.95752	82.58084	5–20	37	0.8	1.3	1.9
483	132	49.95752	82.58084	20–50	41	1.3	1.2	2
484	133	49.8211	82.32539	0–5	18	0.3	0.8	0.8
485	133	49.8211	82.32539	5–20	23	0.9	0.6	0.7
486	133	49.8211	82.32539	20–50	32	1.1	0.4	0.6
